# The Causes of Death and Their Influence in Life Expectancy of Children Aged 5–14 Years in Low- and Middle-Income Countries From 1990 to 2019

**DOI:** 10.3389/fped.2022.829201

**Published:** 2022-05-20

**Authors:** Juanjuan Liang, Yuanze Du, Xiang Qu, Changrong Ke, Guipeng Yi, Mi Liu, Juncheng Lyu, Yanfeng Ren, Jie Xing, Chunping Wang, Shiwei Liu

**Affiliations:** ^1^School of Public Health, Weifang Medical University, Weifang, China; ^2^Hospital Infection Management Office, The Second People's Hospital of Lianyungang, Lianyungang, China; ^3^Tobacco Control Office, Chinese Center for Disease Control and Prevention, Beijing, China

**Keywords:** children and adolescents, cause of death, life expectancy, cause-eliminated life expectancy, low- and middle-income countries

## Abstract

**Introduction:**

Although child and adolescent health is the core of the global health agenda, the cause of death and its expected contribution to life expectancy (LE) among those aged 5–14 are under-researched across countries, especially in low- and middle-income countries (LMICs).

**Methods:**

Death rates per 10 years age group including a 5–14-year-old group were calculated by the formula, which used the population and the number of deaths segmented by the cause of death and gender from the 2019 Global Burden of Disease (GBD) study. LE and cause-eliminated LE in 10-year intervals were calculated by using life tables.

**Results:**

In 2019, the global mortality rate for children and adolescents aged 5–14 years was 0.522 (0.476–0.575) per 1,000, and its LF was 71.377 years. In different-income regions, considerable heterogeneity remains in the ranking of cause of death aged 5–14 years. The top three causes of death in low-income countries (LICs) are enteric infections [0.141 (0.098–0.201) per 1,000], other infectious diseases [0.103 (0.073–0.148) per 1,000], and neglected tropical diseases and malaria [0.102 (0.054–0.172) per 1,000]. Eliminating these mortality rates can increase the life expectancy of the 5–14 age group by 0.085, 0.062, and 0.061 years, respectively. The top three causes of death in upper-middle income countries (upper MICs) are unintentional injuries [0.066 (0.061–0.072) per 1,000], neoplasm [0.046 (0.041–0.050) per 1,000], and transport injuries [0.045 (0.041–0.049) per 1,000]. Eliminating these mortality rates can increase the life expectancy of the 5–14 age group by 0.045, 0.031, and 0.030 years, respectively.

**Conclusion:**

The mortality rate for children and adolescents aged 5–14 years among LMICs remains high. Considerable heterogeneity was observed in the main causes of death among regions. According to the main causes of death at 5–14 years old in different regions and countries at different economic levels, governments should put their priority in tailoring their own strategies to decrease preventable mortality.

## Introduction

Mortality, especially at younger ages, is a key measurement of population health. Avoiding premature mortality from any causes is a crucial goal for every health system, and targets for mortality reduction are central in the development agenda for improving health (1, 2). Mortality rates in children under 5 years old and the variations between countries in the World Health Organization (WHO) Europe are well-studied and documented (3, 4). Mortality in the age group 5–14 is generally the lowest in each age group and is the most vulnerable to be neglected by scholars (5). No studies have provided comprehensive assessments of mortality and life expectancy (LE) in the 5–14-year-old group in different-income regions so far.

Although all-cause mortality at age 5–14 years is low, cause-specific mortality is not necessarily low, such as enteric infections [0.094 (0.067–0.131) per 1,000], unintentional injuries [0.071 (0.060–0.082) per 1,000], and other infectious diseases [0.049 (0.039–0.062) per 1,000], and the health problems of teenagers can also affect the health of adults. For example, adult cardiovascular disease originates from adolescence (6). Risk factors for cardiovascular diseases such as obesity and high blood pressure are spreading among children and adolescents (7), and the case rates are increasing year by year (8, 9). So, it is worth to study the mortality rates in this age group, especially in low- and middle-income countries (LMICs). In 2016, 98% of all deaths of children aged 5–14 years old occurred in LMICs (10). In this article, we aim to identify the main causes of death and the impact of mortality on LE in children and adolescents aged 5–14 years in 137 LMICs and summarize their distribution and trends from 1990 to 2019.

## Methods

### Data Sources

The Global Burden of Disease (GBD) 2019 study provided mortality rate caused by diseases and injuries by age, gender, and location over time and provided the average population by sex and age. We use the Bayesian hierarchical component model to estimate baseline population and net migration at specific ages in 1950 (11, 12). Then, using estimates of fertility, mortality, and net migration from 1950 to 2019 and the 1950 baseline population, fully consistent estimates of the age group population are obtained. We estimated age-specific mortality using data from vital registration systems, sample registration systems, surveys, and censuses (13). Finally, under-5 years old mortality and adult mortality of 15–60 years old were measured based on multiple data sources after adjusting for various data biases using spatiotemporal Gaussian process regression (14–16). Then, the relational model life table system created by the GBD is used to transform the above results into a series of age-specific mortality. Expected mortality rates for each age–sex group were used to estimate the expected life expectancy.

### Geographical Estimates

According to the income levels classified by the World Bank, 137 LMICs were stratified into three groups: 31 low-income countries (LICs), 47 lower middle-income countries (lower MICs), and 59 upper middle-income countries (upper MICs) (Schedule 1).

### Cause of Death Stratification

As in the past, the cause of death in the GBD 2019 has adopted a four-level classification hierarchy to produce mutually exclusive and detailed conclusion. At the GBD hierarchy, the number of mutually exclusive and exhaustive fatal and non-fatal causes at each level estimated by GBD is 3 at level 1, 22 at level 2, 174 at level 3, and 201 at level 4. GBD etiological structure is complete, including the corresponding International Classification of Diseases (ICD)-9 and ICD-10 codes, as well as detailed etiological specific methods [see the corresponding Schedule of the global burden of 369 diseases and injuries in GBD year 2019 (17)]. Considering that the diagnosis level of diseases varies greatly in different regions, the finer the disease classification, the greater the error may be. In addition, due to the low mortality rate in the 5–14 age group, too many classifications of level 3 and level 4 will lead to small differences in mortality from different causes, which is of little significance to the study. So, this study selected 22 causes of death at level 2.

### Statistical Analysis

Data were processed with Excel 2007, and trends in mortality among children aged 5–14 years old were analyzed by using the changing rates from 1990 to 2019. The comparison of mortality rates among children aged 5–14 in different years is expressed as changing rate. Changing rate = (rate for *m* year – rate for *n* year)/rate for *n* year ×100%. Death rate per 10 years was calculated by using the number of dead population and the average population data in the GBD 2019. Cause-eliminated life tables and all-cause life tables of 10-year interval were constructed to assess the contributions of the causes of death. To estimate the number of additional years of life gained because of eliminating a specific cause of death in aged 5–14 years old, each cause of death was eliminated in the 5–14 years old group, not in all age groups to calculate cause-eliminated LE. Variable quantity = LE in *m* year – LE in *n* year. LE loss = cause-eliminated LE – LE, indicating the number of years of LE that the population has been reduced due to the cause of death.

### Patient and Public Involvement

The data in this paper were from the GBD database. Patients and public participants were never involved in this study.

## Results

### Mortality Rate

#### Region and Sex Specific

In 2019, we estimated 677,012 deaths in children aged 5–14 years, and the mortality rate was 0.522 (0.476–0.575) per 1,000 globally. There is a significant difference in mortality rates among different-income regions. The highest mortality rate was 0.898 (0.767–1.057) per 1,000 in LICs, and the lowest was 0.278 (0.261–0.296) per 1,000 in upper MICs, a difference of 2.23 times. The global mortality rates in children aged 5–14 years were 0.568 (0.517–0.627) per 1,000 in boys and 0.473 (0.433–0.519) per 1,000 in girls, and the ratio of boys to girls was 1.20. The boy-to-girl ratio of mortality rates in different-income regions were 1.46 in upper MICs, 1.26 in LICs, and 1.13 in lower MICs (Table 1).

**Table 1:** Changes in sex- and age-specific mortality rates in children aged 5–14 years in specific regions from 1990 to 2019.

		**Global**			**LICs**			**Lower MICs**			**Upper MICs**		
**Year**	**Age group**	**Boy**	**Girl**	**Total**	**Boy**	**Girl**	**Total**	**Boy**	**Girl**	**Total**	**Boy**	**Girl**	**Total**
1990	5–9	1.466 (1.409–1.531)	1.337 (1.294–1.386)	1.403 (1.355–1.459)	2.474 (2.293–2.661)	2.058 (1.918–2.183)	2.269 (2.106–2.425)	1.966 (1.881–2.055)	2.049 (1.966–2.142)	2.006 (1.924–2.095)	1.003 (0.955–1.055)	0.665 (0.639–0.705)	0.839 (0.801–0.882)
	10–14	0.926 (0.894–0.959)	0.757 (0.733–0.783)	0.843 (0.817–0.871)	1.686 (1.563–1.814)	1.291 (1.198–1.378)	1.491 (1.382–1.596)	1.166 (1.121–1.214)	1.105 (1.066–1.151)	1.136 (1.100–1.176)	0.724 (0.683–0.762)	0.457 (0.430–0.495)	0.594 (0.566–0.624)
	5–14	1.208 (1.164–1.255)	1.059 (1.027–1.095)	1.135 (1.098–1.177)	2.115 (1.967–2.273)	1.708 (1.588–1.816)	1.914 (1.777–2.044)	1.594 (1.534–1.661)	1.610 (1.552–1.673)	1.602 (1.544–1.664)	0.866 (0.827–0.906)	0.563 (0.538–0.602)	0.718 (0.687–0.753)
2005	5–9	1.053 (0.988–1.117)	0.931 (0.879–0.983)	0.994 (0.936–1.052)	1.840 (1.688–1.991)	1.546 (1.421–1.654)	1.695 (1.557–1.827)	1.309 (1.221–1.400)	1.253 (1.173–1.331)	1.282 (1.200–1.368)	0.635 (0.606–0.665)	0.420 (0.405–0.436)	0.533 (0.511–0.556)
	10–14	0.747 (0.712–0.783)	0.598 (0.573–0.623)	0.675 (0.645–0.704)	1.312 (1.209–1.407)	1.035 (0.955–1.105)	1.175 (1.081–1.255)	0.947 (0.895–0.998)	0.821 (0.780–0.862)	0.887 (0.844–0.931)	0.488 (0.469–0.508)	0.309 (0.299–0.318)	0.402 (0.389–0.415)
	5–14	0.899 (0.848–0.949)	0.763 (0.725–0.801)	0.834 (0.789–0.877)	1.596 (1.465–1.723)	1.309 (1.205–1.397)	1.454 (1.338–1.560)	1.132 (1.062–1.201)	1.042 (0.986–1.101)	1.089 (1.028–1.153)	0.556 (0.534–0.579)	0.360 (0.349–0.371)	0.462 (0.446–0.479)
2019	5–9	0.621 (0.56–0.694)	0.529 (0.481–0.584)	0.577 (0.522–0.642)	1.120 (0.950–1.344)	1.120 (0.950–1.344)	1.012 (0.856–1.209)	0.726 (0.650–0.807)	0.655 (0.594–0.721)	0.691 (0.623–0.765)	0.332 (0.308–0.356)	0.230 (0.218–0.242)	0.283 (0.266–0.301)
	10–14	0.513 (0.473–0.557)	0.416 (0.383–0.451)	0.466 (0.430–0.506)	0.869 (0.755–1.005)	0.869 (0.755–1.005)	0.771 (0.669–0.887)	0.594 (0.544–0.648)	0.508 (0.466–0.554)	0.553 (0.510–0.598)	0.323 (0.298–0.348)	0.218 (0.205–0.231)	0.273 (0.256–0.291)
	5–14	0.568 (0.517–0.627)	0.473 (0.433–0.519)	0.522 (0.476–0.575)	1.001 (0.861–1.187)	0.792 (0.674–0.923)	0.898 (0.767–1.057)	0.660 (0.600–0.725)	0.582 (0.532–0.637)	0.622 (0.569–0.681)	0.327 (0.305–0.351)	0.224 (0.212–0.236)	0.278 (0.261–0.296)
Change rate (1990–2005)	5–9	−28.172	−30.366	−29.152	−25.627	−24.879	−25.297	−33.418	−38.848	−36.092	−36.690	−36.842	−36.472
	10–14	−19.330	−21.004	−19.929	−22.183	−19.830	−21.194	−18.782	−25.701	−21.919	−32.597	−32.385	−32.323
	5–14	−25.579	−27.951	−26.520	−24.539	−23.361	−24.033	−28.984	−35.280	−32.022	−35.797	−36.057	−35.655
Change rate (2005–2019)	5–9	−41.026	−43.179	−41.952	−39.130	−41.721	−40.295	−44.538	−47.725	−46.100	−47.717	−45.238	−46.904
	10–14	−31.325	−30.435	−30.963	−33.765	−35.266	−34.383	−37.276	−38.124	−37.655	−33.811	−29.450	−32.090
	5–14	−36.819	−38.008	−37.410	−37.281	−39.496	−38.239	−41.696	−44.146	−42.883	−41.187	−37.778	−39.827
Change rate (1990–2019)	5–9	−57.640	−60.434	−58.874	−54.729	−56.220	−55.399	−63.072	−68.033	−65.553	−66.890	−65.414	−66.269
	10–14	−44.600	−45.046	−44.721	−48.458	−48.102	−48.290	−49.057	−54.027	−51.320	−55.387	−52.298	−54.040
	5–14	−52.980	−55.335	−54.009	−52.671	−53.630	−53.083	−58.595	−63.851	−61.174	−62.240	−60.213	−61.281

*LICs, low-income countries; lower MICs, lower middle-income countries; upper MICs, upper middle-income countries*.

#### Age Specific

In 2019, there were 377,740 deaths in children aged 5–9 years old and 299,272 deaths in those aged 10–14 years old globally. Global mortality rates (per 1,000 people) were 0.577 (0.522–0.642) in children aged 5–9 years old and 0.466 (0.430–0.506) in children aged 10–14 years old. The mortality rate in children aged 5–9 years old was higher than that in those aged 10–14 years old, with a ratio of 1.24. The ratios in mortality rates of the two age groups were similar in different-income regions. The ratios of LICs, lower MICs, and upper MICs were 1.31, 1.25, and 1.04, respectively, which showed that the difference in mortality rates between the two age groups is narrower as income levels increase (Table 1).

#### Country Specific

**Figure 1:**
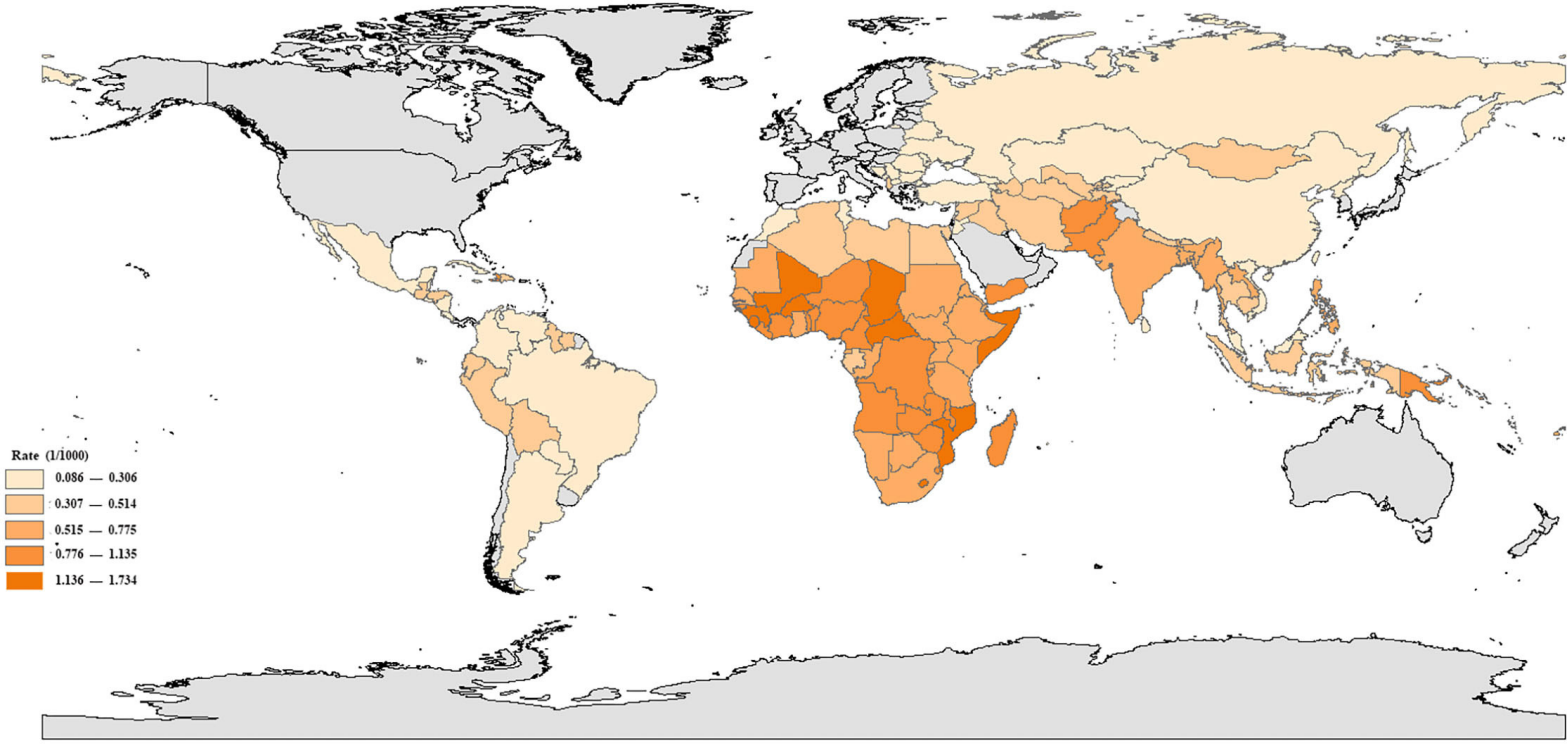
Mortality rates in children aged 5–14 years in 137 low- and middle-income countries, in 2019.

In 2019, mortality rate in children aged 5–14 years old varied greatly between the 137 LMICs (Figure 1). The higher death rates among children and adolescents aged 5–14 were found in Central African Republic [1.734 (1.614–1.935) per 1,000], Somalia [1.416 (1.285–1.541) per 1,000], and Lesotho [1.349 (1.206–1.493) per 1,000] and the lower death rates were found in Serbia [0.086 (0.077–0.097) per 1,000], Montenegro [0.114 (0.099–0.132) per 1,000], and Bosnia and Herzegovina [0.121 (0.103–0.143) per 1,000], with a difference of 19.16 times. The highest ratio for boys and girls in the 137 LMICs was in China 1.72 [0.291 (0.263–0.323) per 1,000 in boys vs. 0.169 (0.156–0.183) per 1,000 in girls] and the lowest was in India 0.94 [0.561 (0.501–0.632) per 1,000 in boys vs. 0.595 (0.538–0.662) per 1,000 in girls] (Schedule 1).

### Changes in Mortality

#### Region Specific

In 2019, the number of deaths in children aged 5–14 years has dropped from 1.274 million in 1990 to 0.677 million globally, decreasing by 0.597 million. Mortality rates also decreased largely by 54.009% from 1990 to 2019 [1.135 (1.098–1.177) per 1,000 in 1990 vs. 0.522 (0.476–0.575) per 1,000 in 2019]. The decrease in mortality rates were similar between the upper MICs [61.281%, 0.718 (0.687–0.753) per 1,000 in 1990 vs. 0.278 (0.261–0.296) per 1,000 in 2019] and the lower MICs [61.174%, 1.602 (1.544–1.664) per 1,000 in 1990 vs. 0.622 (0.569–0.681) per 1,000 in 2019], and slower decreases occurred in LICs [53.083%, 1.914 (1.777–2.044) per 1,000 in 1990 vs. 0.898 (0.767–1.057) per 1,000 in 2019]. Although the decreases of mortality rates for children aged 5–14 years were different in different-income regions, there is homogeneity among them. For example, decreasing rates of different regions in 2005–2019 are all greater than those in 1990–2005 (Table 1).

#### Sex Specific

Globally, the percentage decrease in mortality rates at 5–14 years for girls [55.335%, 1.059 (1.027–1.095) per 1,000 in 1990 vs. 0.473 (0.433–0.519) per 1,000 in 2019] was higher than that for boys [52.980%, 1.208 (1.164–1.255) per 1,000 in 1990 vs. 0.568 (0.517–0.627) per 1,000 in 2019] between 1990 and 2019. In LICs and lower MICs, the percentage decrease in mortality rates for girls was higher than that for boys (Table 1), but in upper MICs, the percentage decrease in mortality rates for boys [62.240%, 0.866 (0.827–0.906) per 1,000 in 1990 vs. 0.327 (0.305–0.351) per 1,000 in 2019] was higher than that for girls [60.213%, 0.563 (0.538–0.602) per 1,000 in 1990 vs. 0.224 (0.212–0.236) per 1,000 in 2019] (Table 1). At the national level, a considerable heterogeneity remains in changing rate in gender. In 57 out of 137 LMICs, the percentage decrease in mortality rates for boys was higher than that for girls. Others were the opposite, with girls higher than boys in the changing rate. The highest boy-to-girl ratio in percentage decrease in mortality rate was in Saint Lucia (31.118% in boys vs. 3.403% in girls), and the lowest was in Fiji (10.688% in boys vs. 25.511% in girls) (Schedule 1).

#### Country Specific

Over the past 30 years, mortality rates in children aged 5–14 years have decreased in 134 countries among 137 LMICs, with higher improvements occurring in Eritrea [79.600%, 3.637 (3.248–4.056) per 1,000 in 1990 vs. 0.742 (0.615–0.934) per 1,000 in 2019], Ethiopia [77.954%, 2.832 (2.689–2.986) per 1,000 in 1990 vs. 0.624 (0.548–0.850) per 1,000 in 2019], and Serbia [76.423%, 0.367 (0.360–0.373) per 1,000 in 1990 vs. 0.086 (0.077–0.097) per 1,000 in 2019] and the lower were in Eswatini [1.764%, 1.071 (0.954–1.184) per 1,000 in 1990 vs. 1.052 (0.827–1.245) per 1,000 in 2019], Fiji [17.511%, 0.642 (0.561–0.741) per 1,000 in 1990 vs. 0.530 (0.450–0.626) per 1,000 in 2019], and Nauru [18.214%, 0.644 (0.578–0.711) per 1,000 in 1990 vs. 0.527 (0.462–0.608) per 1,000 in 2019]. However, in some countries, mortality rates for children aged 5–14 years were increasing. Those countries were Zimbabwe, Lesotho, and Dominica, which have increased by 28.107% [0.867 (0.738–0.953) per 1,000 in 1990 vs. 1.110 (1.002–1.248) per 1,000 in 2019], 14.985% [1.173 (0.866–1.351) per 1,000 in 1990 vs. 1.349 (1.206–1.493) per 1,000 in 2019], and 7.451% [0.400 (0.357–0.440) per 1,000 in 1990 vs. 0.430 (0.361–0.510) per 1,000 in 2019], respectively (Schedule 1).

### Causes of Death

#### Region Specific

In 2019, the global top three causes of death in children aged 5–14 years old were enteric infections [0.094 (0.067–0.131) per 1,000], unintentional injuries [0.071 (0.060–0.082) per 1,000], and other infectious diseases [0.049 (0.039–0.062) per 1,000], accounting for 40.10% of all-cause deaths. In different-income regions, considerable heterogeneity remains in the ranking of causes of death aged 5–14 years old. The top three causes of death in LICs were enteric infections [0.141 (0.098–0.201) per 1,000], other infectious diseases [0.103 (0.073–0.148) per 1,000], and neglected tropical diseases and malaria [0.102 (0.054–0.172) per 1,000]; in lower MICs were enteric infections [0.145 (0.101–0.205) per 1,000], unintentional injuries [0.081 (0.065–0.097) per 1,000], and other infectious diseases [0.063 (0.050–0.078) per 1,000]; and in upper MICs were unintentional injuries [0.066 (0.061–0.072) per 1,000], neoplasm [0.046 (0.041–0.050) per 1,000], and transport injuries [0.045 (0.041–0.049) per 1,000] (Schedule 2).

#### Sex Specific

The global top three causes of death in children aged 5–14 years old for boys were enteric infections [0.098 (0.069–0.141) per 1,000], unintentional injuries [0.089 (0.075–0.104) per 1,000], and transport injuries [0.057 (0.047–0.067) per 1,000] and for girls were enteric infections [0.090 (0.058–0.133) per 1,000], unintentional injuries [0.052 (0.042–0.061) per 1,000], and other infectious diseases [0.049 (0.037–0.063) per 1,000]. Boys have significantly higher mortality in unintentional injuries, transport injuries, neglected tropical diseases and malaria, enteric infections, neoplasm, self-harm, and interpersonal violence than girls, while girls have slightly higher mortality than boys in respiratory infections and tuberculosis and nutritional deficiencies (Schedule 2).

### Changes in Death Sequence

**Figure 2:**
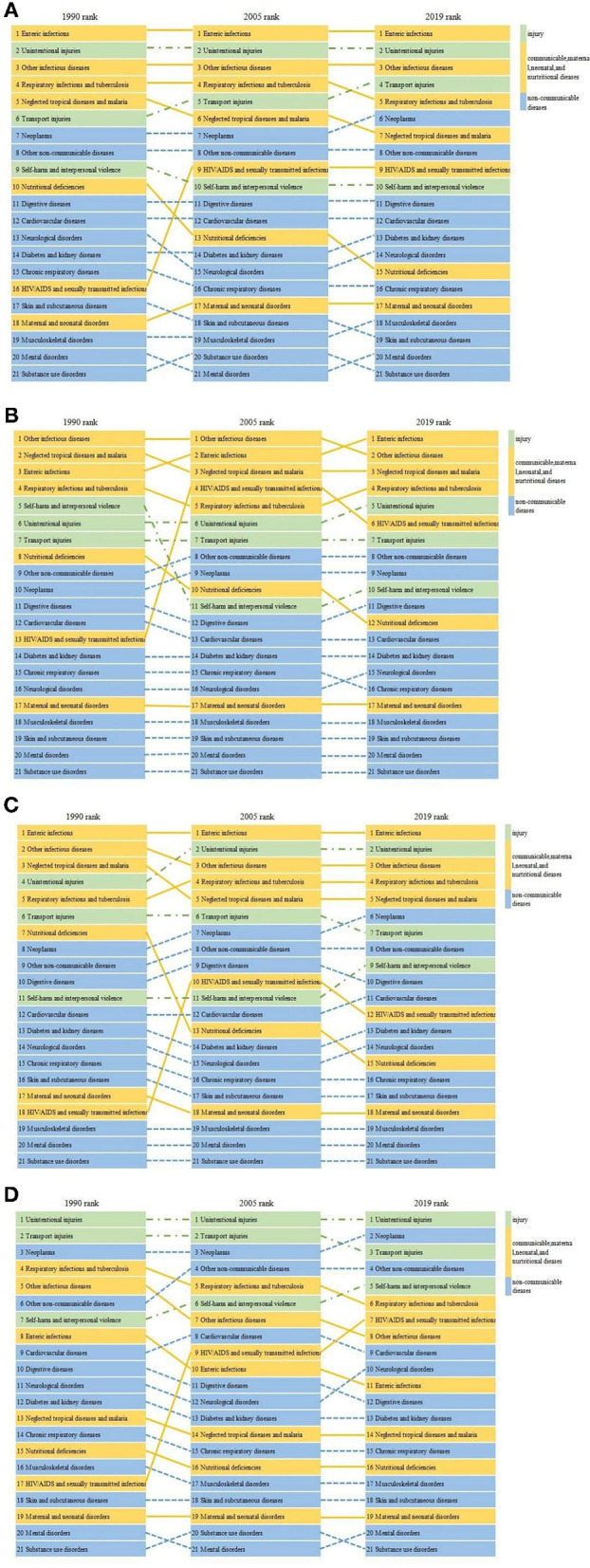
Changes in the rank of causes of death in children aged 5–14 years globally **(A)** and in low-income countries **(B)**, lower middle-income countries **(C)**, and upper middle-income countries **(D)** from 1990 to 2019.

From 1990 to 2019, the global top three causes of death in children aged 5–14 years old remained to be enteric infections, unintentional injuries, and other infectious diseases. The ranking of nutritional deficiencies, neglected tropical diseases and malaria, and skin and subcutaneous diseases have declined, while diseases such as transport injuries, neoplasms, and HIV/AIDS and sexually transmitted diseases have risen. Particularly, HIV/AIDS and sexually transmitted diseases increased from 16th in 1990 to 9th in 2019. Over the past 30 years, the top three causes of death in LICs and upper MICs remained unchanged. However, the top three causes of death in lower MICs have changed from enteric infections, other infectious diseases, neglected tropical diseases, and malaria in 1990 to enteric infections, unintentional injuries, and other infectious diseases in 2019 (Figure 2).

### LE and LE Loss

#### LE

The global LE of 5–14 years old for both sexes combined increased from 66.542 years in 1990 to 68.245 years in 2005 and 71.377 years in 2019. The LE of 5–14 years old remains higher for girls than for boys on a global scale, with 73.970 years for girls and 68.874 years for boys; the absolute increase in the LE of 5–14 years old was 4.822 for boys and 4.823 for girls. In 2019, the LE of 5–14 years in LICs, lower MICs, and upper MICs was 64.808, 68.120, and 72.440 years, respectively. From 1990 to 2019, the LE of 5–14 years old also showed a growth trend in different-income regions, which increased by 6.139 years for LICs, 4.933 years for lower MICs, and 5.275 years for upper MICs. All showed a faster growth during 2005–2019 than 1990–2005. Although in LICs, the LE of 5–14 years was the lowest, the increase in the LE of 5–14 years old was the highest from 1990 to 2019 (Table 2).

**Table 2:** Life expectancy and changes in children aged 5–14 years from 1990 to 2019.

	**Global**			**LICs**			**Lower MICs**			**Upper MICs**		
**Year**	**Boy**	**Girl**	**Total**	**Boy**	**Girl**	**Total**	**Boy**	**Girl**	**Total**	**Boy**	**Girl**	**Total**
1990	64.052	69.147	66.542	56.906	60.586	58.669	61.717	64.832	63.187	64.536	69.937	67.165
2005	65.715	70.909	68.245	58.177	60.652	59.386	63.249	66.968	65.027	65.839	71.900	68.755
2019	68.874	73.970	71.377	62.857	66.792	64.808	66.358	69.962	68.120	69.428	75.627	72.440
Variable quantity (1990–2005)	1.663	1.762	1.703	1.271	0.066	0.717	1.532	2.136	1.840	1.303	1.963	1.590
Variable quantity (2005–2019)	3.159	3.061	3.132	4.680	6.140	5.422	3.109	2.994	3.093	3.589	3.727	3.685
Variable quantity (1990–2019)	4.822	4.823	4.835	5.951	6.206	6.139	4.641	5.130	4.933	4.892	5.690	5.275

*LICs, low-income countries; lower MICs, lower middle-income countries; upper MICs, upper middle-income countries*.

#### LE Loss

In 2019, the global top three major causes of the LE loss in 5–14 years old were as follows: enteric infections (0.063 years), unintentional injuries (0.047 years), and other infectious diseases (0.033 years), resulting in an overall 0.143-year reduction in the LE of this age group and accounting for 41.33% of reduction in the LE of all-cause death. In LICs, the top three causes of LE loss of 5–14 years old were enteric infections (0.085 years), other infectious diseases (0.062 years), and neglected tropical diseases and malaria (0.061 years). In lower MICs, the top three causes of LE loss in 5–14 years old were enteric infections (0.092 years), unintentional injuries (0.051 years), and other infectious diseases (0.040 years). In upper MICs, the top three causes of LE loss in 5–14 years old were unintentional injuries (0.045 years), neoplasms (0.031 years), and transport injuries (0.030 years) (Schedule 3).

#### Changes of the LE Loss

**Figure 3:**
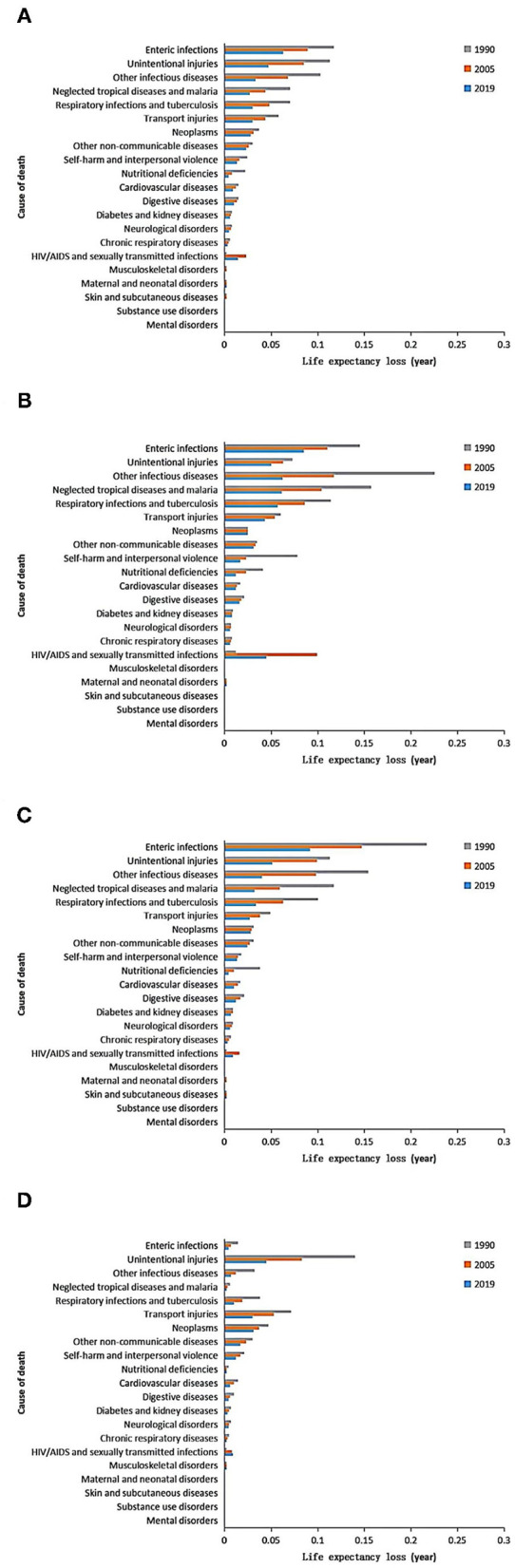
Cause-specific changes of life expectancy (LE) loss in children aged 5–14 years globally **(A)** and in low-income countries **(B)**, lower middle-income countries **(C)**, and upper middle-income countries **(D)** from 1990 to 2019.

Globally, the top three major causes of LE loss in 5–14 years old were stable: enteric infections, unintentional injuries, and other infectious diseases from 1990 to 2019. Except for HIV/AIDS and sexually transmitted infections, LE loss caused by other causes of death were decreased or unchanged (Figure 3). The increase of LE loss caused by HIV/AIDS and sexually transmitted infections in LICs, lower MICs, and upper MICs was 0.033, 0.008, and 0.008 years, respectively. From 1990 to 2019, the changes of LE loss caused by causes of death are heterogeneous in different-income regions. In LICs, the decrease of mortality at age 5–14 years old caused by other infectious diseases, neglected tropical diseases and malaria, and self-harm and interpersonal violence added 0.320 years to the LE in this age group. In lower MICs, the decrease of mortality at age 5–14 years caused by enteric infections, other infectious diseases, and respiratory infections and tuberculosis added 0.305 years to the LE in this age group. In upper MICs, the decrease of mortality at age 5–14 years caused by unintentional injuries, respiratory infections and tuberculosis, and transport injuries added 0.164 years to the LE in this age group (Schedule 3).

## Discussion

### Main Findings

Since 1990, despite the frequency of catastrophic events, such as the genocide in Rwanda (1994), the war in Syria, and the increase in mortality caused by HIV/AIDS in some areas (15), the global mortality rate in children aged 5–14 years old was declining. This is mainly due to the significant decrease in mortality rates caused by other infectious diseases, unintentional injuries, enteric infections, neglected tropical diseases and malaria, and respiratory infections and tuberculosis. From 1900 to 2019, the ranking of death causes in children aged 5–14 years old has changed significantly. The ranking of nutritional deficiencies, neglected tropical diseases and malaria, and respiratory infections and tuberculosis have declined, while diseases such as transport injuries, neoplasms, and HIV/AIDS and sexually transmitted diseases have risen. HIV/AIDS and sexually transmitted diseases increased from 16th in 1990 to 9th in 2019.

In 2019, the global LE of children aged 5–14 years is 71.38 years, and the top three causes of loss of years of LE in this age group are enteric infections, unintentional injuries, and other infectious diseases. In different-income regions, the main causes that had the biggest impact on LE were different. In LICs, the top three causes of LE loss were enteric infections, other infectious diseases, and neglected tropical diseases and malaria. In lower MICs, the top three causes were enteric infections, unintentional injuries, and other infectious diseases. In upper MICs, the top three causes were unintentional injuries, neoplasms, and transport injuries. Over the past 30 years, the global LE of children aged 5–14 years old has generally increased steadily, from 66.542 years in 1990 to 71.377 years in 2019. Except for HIV/AIDS and sexually transmitted infections, LE loss caused by other causes of death was decreased or unchanged.

### Mortality

Over the past 30 years, mortality rates in children aged 5–14 years have declined substantially, with rates of change accelerating globally since 2005, particularly compared with the previous 15 years, 1990–2005. This finding is continued evidence that effective progress is being made in tackling the main causes of children's death, which is linked to the right strategies and measures, including expanded vaccination programs (18, 19), improved water and sanitation (20, 21), increased income per capita (22), improving educational levels of mothers (23, 24), declining levels of fertility (25, 26), and a range of health programs expanding health development assistance (27). We found that the mortality rates of age 5–14 years old were higher for boys than for girls. Boys' mortality rates caused by accidental injuries and traffic injuries were significantly higher than girls. This could be related to boys who are extroverted, active, more willing to try the dangerous game and new things, often adventurous but unaware of dangerous things, and more exposed to risk factors than girls (28, 29).

Mortality rates of age 5–14 years old varied considerably in different-income regions. The mortality rate in LICs is 3.23 times that of upper MICs, with higher mortality rate caused by enteric infections, neglected tropical diseases and malaria, other infectious diseases, respiratory infections and tuberculosis, and HIV/AIDS and sexually transmitted infections. In addition, mortality rates varied considerably across 137 LMICs, with the Central African Republic 20.07 times that of Serbia. It was suggested that achieving sustainability in reducing child mortality required a major shift in resource allocation and that support for low-income countries should be prioritized. Strengthening funding for basic public health services, expanding women's education, and continuing to innovate interventions to reduce child mortality are likely to be key components in achieving sustainable development goals to reduce child mortality.

### Life Expectancy

LE is one of the most important indexes to evaluate the health status of the population, which reflects the average number of years a particular population can continue to live, and its change over time can be used as a public health index to evaluate the social and economic conditions and the level of health care development (30). There was an obvious gender discrepancy and geographical inequality in LE. This study found that in 2019, global LE of children aged 5–14 years was 71.377 years, and the girls' (73.970 years) had higher global LE than the boys' (68.874 years). It has been suggested that the difference in the LE between boys and girls could be partly related to biological factors (31). Also, gender-related factors such as risky behavior of men, differences in body size, hormonal factors, and innate immunity could contribute to the observed differences in human mortality (29, 32). However, greater differences in LE are also considered related to environmental factors (33). Between 1990 and 2019, the global LE of children aged 5–14 years increased by 4.835 years, while that of LICs, lower MICs, and upper MICs increased by 6.39, 4.933, and 5.275 years, respectively. The speed of development of LE is affected by various factors. Large variations can be attributed to different economic situations, nutritional and lifestyle factors, work-related and social factors, as well as public health strategies and medical care (34).

### Main Causes of Death

We found that the influence of a cause on mortality was proportional to its influence on LE. In 2019, although enteric infections, unintentional injuries, and other infectious diseases are the leading causes of death among children aged 5–14 years globally, they are also the three diseases that decreased most dramatically in that age group during 1990–2019, which makes the greatest contribution (53.06%) to the total decline. Reducing child mortality from enteric infections, unintentional injuries, and other infectious diseases is the key to reduce mortality and increase LE in this age group in the future. In addition, it is worth noting that the mortality caused by HIV/AIDS and sexually transmitted infections increased from 0.003 (0.002–0.004) per 1,000 in 1990 to 0.022 (0.018–0.026) per 1,000 in 2019, which is the only cause contributing to the increase in mortality among the 21 causes of death. There are three ways of transmission of AIDS, mother-to-child transmission, sexual transmission, and blood transmission. The route of HIV transmission in children varies according to age. The majority of HIV-infected newborns and children are infected through mother-to-child transmission, mainly during intrauterine delivery and delivery and also through breastfeeding after delivery, and a small part of the transmission is through blood products or other close contact (35, 36). HIV in adolescents, who are in the period of sexual development and psychological formation, can also be transmitted through sexual contact (37) or intravenous drug abuse. Some precautions should be done to reduce the prevalence and mortality of HIV/AIDS in children, such as increasing the strength of AIDS test for females before marriage and pregnancy, carrying out effective mother–child blocking work, strengthening sex education for teenagers, and timely detection and treatment of children with AIDS. Facing the urge to eliminate HIV/AIDS by 2030, more rapid progress is needed (38, 39). However, stagnating assistance for HIV/AIDS programs amplifies the challenge of reducing HIV/AIDS mortality (40, 41).

### Limitations

The GBD study has some limitations, which have been described elsewhere (13, 14). Some of the most important ones may be included in this study. In view of the scarcity of accurate data in most low-income and middle-income countries, which is attributable to the absence of adequate civil registration systems, our estimates of death rates at ages 5–9 and 10–14 years in settings depend on the selection of a model life table reference using the under-five death rates, the adult mortality rate, and the database of empirical mortality patterns in the life table database. Compared with infant mortality, young adult mortality has a weak response to the epidemiological transition; in addition, the model fitting of child and adult mortality estimates usually comes from different data sources, and there may be different deviations; therefore, the simulation of 5–14 years old child mortality estimates may have a serious distortion. For example, the data from this study indicate that the largest cause of death in this age group is intestinal disease, but the clinical fact is that deaths from intestinal diseases outside infants is extremely rare. More scientific methods of mortality estimation should be explored in the future, and the collection of basic data should be strengthened, especially in low- and middle-income areas. It is calling for the expansion of global policy focus on children and adolescents aged 5–14 and the strengthening of relevant research in the 5–14 age group, which was the original purpose of this study.

## Conclusion

In summary, from 2000 to 2019, the mortality rates of children and adolescents aged 5–14 years old showed a decreasing trend, and the LE of this age group showed an increasing trend. Except for HIV/AIDS and sexually transmitted infections, loss of LE and mortality rates of other causes of death were decreased or unchanged. In different-income regions, the main causes of death and the loss of LE in children aged 5–14 years old remain considerably heterogeneous. In LICs, the top three causes of death and loss of LE in age 5–14 years old were enteric infection, other infectious diseases, and neglected tropical diseases and malaria; in lower MICs, they were enteric infections, unintentional injuries, and other infectious diseases; and in upper MICs, they were unintentional injuries, neoplasm, and transport injuries. Our findings can serve as a useful reference to inform targeted strategies to decrease preventable mortality at regional levels.

## Data Availability Statement

The original contributions presented in the study are included in the article/Supplementary Material, further inquiries can be directed to the corresponding author/s.

## Author Contributions

SL and CW designed the research and had final responsibility for the decision to submit for publication. JLi and JX completed the data analysis and drafting of the manuscript. CK, GY, and XQ provided technical and material support. ML, JLy, and YR collected the data. YD made significant contributions to the content and data modification in the process of revision. All authors contributed to the article and approved the submitted version.

## Funding

This work was supported by the National Natural Science Foundation of China (71673202) and the Shandong Provincial Humanities and Social Science Project in 2020 (2020-NDGL-30).

## Conflict of Interest

The authors declare that the research was conducted in the absence of any commercial or financial relationships that could be construed as a potential conflict of interest.

## Publisher's Note

All claims expressed in this article are solely those of the authors and do not necessarily represent those of their affiliated organizations, or those of the publisher, the editors and the reviewers. Any product that may be evaluated in this article, or claim that may be made by its manufacturer, is not guaranteed or endorsed by the publisher.
