# Supplementary material for: The Causes of Death and Their Influence in Life Expectancy of Children Aged 5–14 Years in Low- and Middle-Income Countries From 1990 to 2019
Source: Front Pediatr. 2022 May 20;10:829201. doi: 10.3389/fped.2022.829201 (PMC9164626; doi:10.3389/fped.2022.829201)
Supplement: Supplementary file 1 [file Data_Sheet_1.pdf]

Geographical estimates

According to the income levels classified by the World Bank ,137 low-and middle-income countries were stratified into three groups: 31 low-income countries (LICs), 47 lower-middle income countries (Lower MICs) and 59 upper-middle income countries (Upper MICs):

31 LICs: Afghanistan, Benin, Burkina Faso, Burundi, Central African Republic, Chad, Democratic People's Republic of Korea, Democratic Republic of the Congo, Eritrea, Ethiopia, Gambia, Guinea, Guinea-Bissau, Haiti, Liberia, Madagascar, Malawi, Mali, Mozambique, Nepal, Niger, Rwanda, Sierra Leone, Somalia, South Sudan, Syrian Arab Republic, Tajikistan, Togo, Uganda, United Republic of Tanzania, Yemen.

47 Lower MICs: Angola, Bangladesh, Bhutan, Bolivia (Plurinational State of), Cabo Verde, Cambodia, Cameroon, Comoros, Congo, Côte d'Ivoire, Djibouti, Egypt, El Salvador, Eswatini, Ghana, Honduras, India, Indonesia, Kenya, Kiribati, Kyrgyzstan, Lao People's Democratic Republic, Lesotho, Mauritania, Micronesia (Federated States of), Mongolia, Morocco, Myanmar, Nicaragua, Nigeria, Pakistan, Palestine, Papua New Guinea, Philippines, Republic of Moldova, Sao Tome and Principe, Senegal, Solomon Islands, Sudan, Timor-Leste, Tunisia, Ukraine, Uzbekistan, Vanuatu, Viet Nam, Zambia, Zimbabwe.

59 Upper MICs: Albania, Algeria, American Samoa, Argentina, Armenia, Azerbaijan, Belarus, Belize, Bosnia and Herzegovina, Botswana, Brazil, Bulgaria, China, Colombia, Costa Rica, Cuba, Dominica, Dominican Republic, Ecuador, Equatorial Guinea, Fiji, Gabon, Georgia, Grenada, Guatemala, Guyana, Iran (Islamic Republic of), Iraq, Jamaica, Jordan, Kazakhstan, Lebanon, Libya, Malaysia, Maldives, Marshall Islands, Mauritius, Mexico, Montenegro, Namibia, Nauru, North Macedonia, Paraguay, Peru, Romania, Russian Federation, Saint Lucia, Saint Vincent and the Grenadines, Samoa, Serbia, South Africa, Sri Lanka, Suriname, Thailand, Tonga, Turkey, Turkmenistan, Tuvalu, Venezuela (Bolivarian Republic of).

Schedule 1 Mortality and its change rate in children age 5-14 years in 137 low- and middle-income countries from 1990 to 2019

	1990 year			2005 year			2019 year			change rate（1990-2005）			change rate（2005-2019）			change rate（1990-2019）		
	male	female	total	male	female	total	male	female	total	male	female	total	male	female	total	male	female	total
**Country-specific**																		
**31 LICs**																		
Afghanistan	1.966<br>(1.688-2.193)	1.830<br>(1.628-1.986)	1.899<br>(1.655-2.089)	1.544<br>(1.400-1.687)	1.377<br>(1.204-1.519)	1.463<br>(1.312-1.601)	0.882<br>(0.769-1.033)	0.746<br>(0.646-0.964)	0.816<br>(0.711-0.997)	-21.443	-24.724	-22.970	-42.879	-45.825	-44.215	-55.127	-59.219	-57.029
Benin	1.743<br>(1.519-1.973)	1.384<br>(1.159-1.602)	1.571<br>(1.344-1.793)	1.417<br>(1.144-1.722)	1.122<br>(0.857-1.404)	1.272<br>(1.007-1.567)	1.093<br>(0.785-1.576)	0.861<br>(0.580-1.234)	0.978<br>(0.683-1.407)	-18.739	-18.962	-18.999	-22.870	-23.268	-23.156	-37.323	-37.817	-37.755
Burkina Faso	1.964<br>(1.708-2.222)	1.556<br>(1.299-1.817)	1.764<br>(1.510-2.020)	1.779<br>(1.507-2.036)	1.470<br>(1.193-1.706)	1.627<br>(1.352-1.869)	1.288<br>(1.001-1.630)	1.036<br>(0.766-1.339)	1.164<br>(0.885-1.485)	-9.420	-5.537	-7.758	-27.590	-29.551	-28.465	-34.411	-33.451	-34.014
Burundi	2.433<br>(2.189-2.643)	1.913<br>(1.720-2.060)	2.172<br>(1.967-2.349)	2.923<br>(2.625-3.191)	2.588<br>(2.373-2.786)	2.751<br>(2.494-2.975)	1.178<br>(0.943-1.298)	0.944<br>(0.803-1.003)	1.059<br>(0.873-1.134)	20.111	35.280	26.661	-59.699	-63.516	-61.498	-51.594	-50.645	-51.233
Central African Republic	2.552<br>(2.356-2.764)	1.844<br>(1.623-2.032)	2.202<br>(2.020-2.393)	2.774<br>(2.518-3.058)	1.997<br>(1.832-2.171)	2.386<br>(2.206-2.579)	2.068<br>(1.862-2.414)	1.400<br>(1.329-1.472)	1.734<br>(1.614-1.935)	8.726	8.282	8.337	-25.445	-29.883	-27.314	-18.939	-24.076	-21.254
Chad	1.973<br>(1.727-2.188)	1.639<br>(1.400-1.837)	1.806<br>(1.561-2.011)	1.923<br>(1.688-2.143)	1.625<br>(1.400-1.803)	1.774<br>(1.546-1.969)	1.456<br>(1.074-1.807)	1.218<br>(0.841-1.483)	1.338<br>(0.956-1.649)	-2.534	-0.849	-1.757	-24.309	-25.036	-24.596	-26.227	-25.673	-25.921
Democratic People's Republic of Korea	0.779<br>(0.604-1.092)	0.603<br>(0.438-0.888)	0.691<br>(0.522-0.992)	0.549<br>(0.481-0.622)	0.380<br>(0.330-0.427)	0.467<br>(0.406-0.528)	0.320<br>(0.274-0.377)	0.211<br>(0.187-0.243)	0.267<br>(0.232-0.312)	-29.589	-36.910	-32.472	-41.718	-44.534	-42.847	-58.963	-65.006	-61.406
Democratic Republic of the Congo	1.876<br>(1.528-2.205)	1.519<br>(1.188-1.760)	1.698<br>(1.357-1.985)	1.782<br>(1.553-2.005)	1.461<br>(1.278-1.614)	1.622<br>(1.420-1.809)	1.015<br>(0.797-1.223)	0.802<br>(0.618-0.965)	0.909<br>(0.708-1.096)	-4.990	-3.850	-4.457	-43.073	-45.069	-43.972	-45.913	-47.184	-46.469
Eritrea	3.920<br>(3.532-4.341)	3.337<br>(2.952-3.772)	3.637<br>(3.248-4.056)	1.419<br>(1.248-1.633)	0.966<br>(0.882-1.063)	1.197<br>(1.073-1.354)	0.886<br>(0.715-1.134)	0.592<br>(0.506-0.731)	0.742<br>(0.615-0.934)	-63.800	-71.043	-67.081	-37.528	-38.686	-38.032	-77.386	-82.245	-79.600
Ethiopia	3.121<br>(2.943-3.313)	2.522<br>(2.393-2.639)	2.832<br>(2.689-2.986)	1.656<br>(1.536-1.773)	1.380<br>(1.282-1.482)	1.522<br>(1.415-1.628)	0.711<br>(0.635-0.909)	0.533<br>(0.447-0.779)	0.624<br>(0.548-0.850)	-46.947	-45.258	-46.272	-57.042	-61.390	-58.968	-77.210	-78.864	-77.954
Gambia	1.392<br>(1.036-1.717)	1.081<br>(0.763-1.309)	1.237<br>(0.896-1.509)	1.164<br>(0.947-1.316)	0.952<br>(0.787-1.064)	1.057<br>(0.866-1.187)	0.674<br>(0.580-0.792)	0.507<br>(0.438-0.603)	0.590<br>(0.509-0.697)	-16.398	-11.965	-14.520	-42.104	-46.789	-44.178	-51.598	-53.155	-52.283
Guinea	2.133<br>(1.901-2.315)	1.853<br>(1.602-2.028)	1.996<br>(1.760-2.176)	1.723<br>(1.499-1.951)	1.437<br>(1.233-1.624)	1.580<br>(1.363-1.782)	1.452<br>(1.102-1.839)	1.210<br>(0.919-1.482)	1.333<br>(1.013-1.658)	-19.210	-22.442	-20.869	-15.716	-15.805	-15.633	-31.907	-34.700	-33.239
Guinea-Bissau	2.766<br>(2.332-3.206)	1.986<br>(1.748-2.205)	2.379<br>(2.048-2.705)	2.140<br>(1.895-2.370)	1.609<br>(1.479-1.740)	1.876<br>(1.689-2.053)	1.266<br>(1.181-1.437)	0.931<br>(0.878-0.986)	1.100<br>(1.040-1.200)	-22.637	-18.972	-21.148	-40.834	-42.180	-41.368	-54.228	-53.150	-53.768
Haiti	1.846<br>(1.649-2.040)	1.553<br>(1.429-1.674)	1.697<br>(1.541-1.846)	1.439<br>(1.284-1.577)	1.282<br>(1.161-1.402)	1.361<br>(1.226-1.481)	1.222<br>(1.004-1.403)	1.026<br>(0.795-1.170)	1.125<br>(0.912-1.288)	-22.038	-17.437	-19.801	-15.120	-19.974	-17.371	-33.826	-33.928	-33.732
Liberia	3.016<br>(2.720-3.274)	2.667<br>(2.374-2.903)	2.842<br>(2.544-3.081)	1.400<br>(1.208-1.554)	1.245<br>(1.058-1.372)	1.323<br>(1.134-1.462)	0.990<br>(0.691-1.177)	0.884<br>(0.567-1.036)	0.938<br>(0.628-1.104)	-53.572	-53.324	-53.441	-29.312	-28.963	-29.130	-67.181	-66.843	-67.004
Madagascar	2.881	2.358	2.621	1.376	1.174	1.275	0.949	0.799	0.875	-52.263	-50.215	-51.338	-31.022	-31.929	-31.406	-67.072	-66.111	-66.621

	(2.633-3.125)	(2.194-2.525)	(2.417-2.822)	(1.233-1.507)	(1.072-1.263)	(1.154-1.384)	(0.762-1.134)	(0.603-0.975)	(0.685-1.052)									
Malawi	2.165 (1.907-2.369)	1.745 (1.486-1.888)	1.954 (1.697-2.131)	1.984 (1.573-2.282)	1.586 (1.196-1.833)	1.783 (1.386-2.052)	1.063 (0.884-1.351)	0.784 (0.627-1.022)	0.922 (0.756-1.184)	-8.342	-9.152	-8.776	-46.444	-50.536	-48.270	-50.912	-55.063	-52.810
Mali	2.314 (2.164-2.473)	2.052 (1.913-2.195)	2.185 (2.044-2.332)	1.668 (1.414-1.879)	1.408 (1.153-1.616)	1.540 (1.284-1.749)	1.324 (0.957-1.862)	1.092 (0.755-1.547)	1.210 (0.857-1.706)	-27.893	-31.394	-29.535	-20.635	-22.395	-21.426	-42.772	-46.758	-44.633
Mozambique	2.172 (1.912-2.394)	1.732 (1.481-1.928)	1.951 (1.699-2.162)	1.773 (1.451-2.129)	1.381 (1.074-1.693)	1.577 (1.264-1.914)	1.446 (1.142-1.769)	1.115 (0.868-1.404)	1.279 (1.002-1.585)	-18.367	-20.288	-19.208	-18.459	-19.261	-18.866	-33.436	-35.642	-34.450
Nepal	1.518 (1.309-1.737)	1.391 (1.182-1.569)	1.456 (1.244-1.655)	0.753 (0.670-0.936)	0.631 (0.541-0.840)	0.693 (0.609-0.888)	0.485 (0.424-0.545)	0.369 (0.316-0.421)	0.428 (0.372-0.484)	-50.412	-54.679	-52.438	-35.580	-41.524	-38.201	-68.055	-73.498	-70.607
Niger	2.442 (2.144-2.781)	1.967 (1.709-2.289)	2.203 (1.926-2.533)	1.714 (1.468-1.969)	1.414 (1.174-1.668)	1.567 (1.323-1.817)	1.239 (0.887-1.767)	1.028 (0.698-1.473)	1.135 (0.794-1.621)	-29.827	-28.114	-28.894	-27.718	-27.296	-27.589	-49.277	-47.736	-48.511
Rwanda	2.533 (2.381-2.690)	2.045 (1.911-2.154)	2.286 (2.146-2.416)	1.496 (1.236-1.711)	1.222 (0.994-1.384)	1.357 (1.110-1.542)	0.685 (0.565-0.923)	0.515 (0.395-0.733)	0.600 (0.482-0.831)	-40.954	-40.218	-40.636	-54.208	-57.848	-55.792	-72.962	-74.801	-73.756
Sierra Leone	2.070 (1.770-2.476)	1.574 (1.341-1.950)	1.819 (1.552-2.213)	1.964 (1.756-2.185)	1.633 (1.453-1.806)	1.798 (1.601-1.993)	1.372 (0.907-1.741)	1.158 (0.728-1.414)	1.265 (0.818-1.577)	-5.164	3.697	-1.150	-30.100	-29.074	-29.669	-33.710	-26.452	-30.478
Somalia	2.669 (2.296-2.955)	2.111 (1.881-2.299)	2.413 (2.114-2.651)	2.068 (1.760-2.328)	1.585 (1.411-1.758)	1.839 (1.605-2.045)	1.601 (1.412-1.840)	1.217 (1.153-1.260)	1.416 (1.285-1.541)	-22.508	-24.917	-23.782	-22.590	-23.211	-23.016	-40.014	-42.345	-41.324
South Sudan	1.792 (1.449-2.188)	1.341 (1.054-1.706)	1.578 (1.259-1.953)	1.291 (1.062-1.701)	0.974 (0.772-1.363)	1.142 (0.923-1.547)	1.055 (0.882-1.503)	0.801 (0.653-1.196)	0.934 (0.773-1.359)	-27.954	-27.401	-27.609	-18.259	-17.754	-18.188	-41.109	-40.290	-40.775
Syrian Arab Republic	0.950 (0.853-1.058)	0.708 (0.646-0.777)	0.832 (0.754-0.919)	0.500 (0.479-0.525)	0.386 (0.37-0.402)	0.445 (0.431-0.459)	0.518 (0.459-0.584)	0.388 (0.351-0.428)	0.454 (0.408-0.507)	-47.302	-45.510	-46.499	3.513	0.451	2.067	-45.450	-45.264	-45.394
Tajikistan	0.843 (0.789-0.905)	0.588 (0.558-0.622)	0.717 (0.676-0.763)	0.717 (0.666-0.772)	0.473 (0.445-0.502)	0.597 (0.559-0.639)	0.460 (0.411-0.516)	0.318 (0.291-0.350)	0.391 (0.353-0.436)	-14.960	-19.538	-16.690	-35.886	-32.810	-34.565	-45.477	-45.938	-45.486
Togo	1.590 (1.346-1.798)	1.300 (1.064-1.479)	1.447 (1.203-1.638)	1.588 (1.203-1.866)	1.248 (0.898-1.459)	1.424 (1.059-1.668)	1.034 (0.758-1.301)	0.796 (0.543-0.987)	0.919 (0.654-1.152)	-0.131	-4.014	-1.549	-34.853	-36.241	-35.506	-34.938	-38.801	-36.505
Uganda	1.754 (1.505-2.104)	1.295 (1.058-1.611)	1.524 (1.282-1.850)	1.893 (1.540-2.153)	1.501 (1.186-1.694)	1.697 (1.365-1.923)	0.902 (0.772-1.188)	0.642 (0.527-0.894)	0.775 (0.654-1.045)	7.963	15.907	11.360	-52.371	-57.233	-54.345	-48.578	-50.430	-49.158
United Republic of Tanzania	1.499 (1.271-1.729)	1.198 (0.986-1.415)	1.348 (1.128-1.575)	1.246 (1.098-1.513)	1.025 (0.879-1.290)	1.135 (0.989-1.398)	0.810 (0.677-1.086)	0.657 (0.511-0.919)	0.733 (0.595-1.001)	-16.858	-14.424	-15.776	-34.999	-35.886	-35.401	-45.957	-45.133	-45.592
Yemen	1.445 (1.107-1.787)	1.235 (0.886-1.524)	1.345 (1.009-1.661)	0.928 (0.681-1.191)	0.800 (0.537-1.022)	0.866 (0.613-1.108)	0.875 (0.733-1.070)	0.766 (0.622-0.957)	0.822 (0.679-1.017)	-35.744	-35.223	-35.612	-5.762	-4.249	-5.103	-39.446	-37.975	-38.897
**47 Lower MICs**																		
Angola	2.554 (2.183-2.934)	2.023 (1.72-2.268)	2.288 (1.947-2.605)	1.771 (1.518-2.016)	1.410 (1.219-1.563)	1.588 (1.366-1.786)	0.958 (0.651-1.11)	0.728 (0.462-0.845)	0.842 (0.555-0.973)	-30.655	-30.277	-30.587	-45.874	-48.356	-46.968	-62.467	-63.992	-63.189
Bangladesh	2.604 (2.471-2.737)	2.368 (2.264-2.489)	2.489 (2.378-2.615)	1.349 (1.263-1.434)	1.181 (1.112-1.251)	1.268 (1.192-1.343)	0.764 (0.677-0.856)	0.616 (0.553-0.683)	0.691 (0.616-0.769)	-48.196	-50.115	-49.053	-43.353	-47.892	-45.549	-70.654	-74.006	-72.259
Bhutan	1.305 (1.044-1.566)	1.213 (0.912-1.456)	1.261 (0.983-1.513)	0.641 (0.573-0.784)	0.536 (0.465-0.702)	0.589 (0.519-0.745)	0.440 (0.378-0.536)	0.344 (0.292-0.44)	0.392 (0.335-0.487)	-50.847	-55.847	-53.308	-31.361	-35.730	-33.368	-66.262	-71.623	-68.889
Bolivia (Plurinational State of)	1.258 (1.101-1.403)	1.094 (0.932-1.211)	1.177 (1.018-1.305)	0.709 (0.580-0.893)	0.624 (0.477-0.813)	0.667 (0.530-0.855)	0.473 (0.408-0.541)	0.375 (0.323-0.428)	0.425 (0.365-0.485)	-43.598	-43.023	-43.298	-33.311	-39.845	-36.328	-62.386	-65.725	-63.897
Cabo Verde	0.634 (0.612-0.657)	0.613 (0.595-0.633)	0.624 (0.604-0.643)	0.557 (0.506-0.613)	0.412 (0.383-0.444)	0.485 (0.445-0.528)	0.379 (0.342-0.420)	0.231 (0.211-0.258)	0.306 (0.277-0.339)	-12.160	-32.835	-22.254	-31.907	-43.825	-36.914	-40.187	-62.269	-50.954
Cambodia	1.970 (1.797-2.122)	1.590 (1.473-1.693)	1.781 (1.636-1.907)	1.139 (0.983-1.265)	0.884 (0.778-0.965)	1.015 (0.884-1.115)	0.565 (0.495-0.651)	0.389 (0.335-0.453)	0.480 (0.417-0.554)	-42.192	-44.371	-43.022	-50.380	-55.968	-52.746	-71.316	-75.505	-73.076
Cameroon	1.564 (1.385-1.739)	1.284 (1.105-1.424)	1.427 (1.250-1.581)	1.674 (1.373-1.892)	1.364 (1.087-1.526)	1.522 (1.235-1.709)	1.090 (0.796-1.393)	0.886 (0.611-1.131)	0.989 (0.707-1.266)	7.043	6.282	6.656	-34.920	-35.090	-35.003	-30.337	-31.013	-30.678
Comoros	1.584 (1.313-1.842)	1.322 (1.100-1.498)	1.455 (1.208-1.671)	1.123 (0.888-1.307)	0.994 (0.748-1.141)	1.059 (0.817-1.224)	0.765 (0.572-0.979)	0.669 (0.458-0.856)	0.718 (0.515-0.918)	-29.073	-24.873	-27.190	-31.891	-32.671	-32.239	-51.692	-49.418	-50.663

Congo	1.551 (1.421-1.693)	1.122 (1.024-1.270)	1.334 (1.225-1.465)	1.579 (1.476-1.690)	1.312 (1.217-1.406)	1.445 (1.346-1.540)	0.802 (0.680-0.929)	0.590 (0.508-0.664)	0.696 (0.594-0.799)	1.824	16.947	8.275	-49.192	-55.009	-51.821	-48.265	-47.385	-47.834
Côte d’Ivoire	1.615 (1.342-1.883)	1.239 (0.971-1.464)	1.431 (1.161-1.674)	1.887 (1.461-2.210)	1.515 (1.125-1.769)	1.705 (1.299-2.000)	1.084 (0.817-1.407)	0.865 (0.618-1.130)	0.978 (0.719-1.274)	16.841	22.249	19.205	-42.583	-42.921	-42.638	-32.913	-30.222	-31.621
Djibouti	1.395 (1.020-1.750)	1.117 (0.784-1.380)	1.267 (0.910-1.580)	1.130 (0.777-1.500)	0.901 (0.569-1.193)	1.028 (0.685-1.362)	0.808 (0.611-1.062)	0.630 (0.432-0.851)	0.727 (0.531-0.966)	-18.998	-19.318	-18.899	-28.558	-30.094	-29.220	-42.131	-43.598	-42.596
Egypt	1.474 (1.386-1.566)	1.250 (1.182-1.319)	1.365 (1.287-1.444)	0.742 (0.685-0.806)	0.515 (0.480-0.555)	0.632 (0.587-0.684)	0.448 (0.373-0.536)	0.313 (0.271-0.363)	0.383 (0.324-0.453)	-49.639	-58.773	-53.702	-39.674	-39.224	-39.412	-69.619	-74.944	-71.949
El Salvador	0.933 (0.883-0.985)	0.767 (0.730-0.804)	0.851 (0.809-0.895)	0.503 (0.465-0.541)	0.379 (0.357-0.401)	0.442 (0.412-0.472)	0.323 (0.269-0.391)	0.240 (0.207-0.282)	0.283 (0.238-0.338)	-46.037	-50.542	-48.017	-35.736	-36.690	-36.064	-65.322	-68.688	-66.764
Eswatini	1.275 (1.140-1.405)	0.871 (0.746-0.970)	1.071 (0.954-1.184)	1.599 (1.358-1.851)	0.971 (0.765-1.175)	1.28 (1.059-1.509)	1.252 (0.978-1.470)	0.837 (0.668-1.012)	1.052 (0.827-1.245)	25.443	11.502	19.487	-21.701	-13.786	-17.785	-1.779	-3.870	-1.764
Ghana	1.467 (1.206-1.710)	1.238 (0.982-1.420)	1.355 (1.101-1.566)	1.380 (1.172-1.523)	1.122 (0.933-1.227)	1.252 (1.053-1.374)	0.856 (0.669-1.069)	0.653 (0.471-0.847)	0.756 (0.573-0.955)	-5.920	-9.350	-7.538	-37.992	-41.843	-39.650	-41.663	-47.281	-44.199
Honduras	1.186 (1.101-1.278)	1.134 (1.062-1.208)	1.161 (1.084-1.240)	0.720 (0.651-0.798)	0.653 (0.599-0.712)	0.687 (0.625-0.757)	0.375 (0.296-0.446)	0.281 (0.250-0.320)	0.329 (0.274-0.384)	-39.318	-42.467	-40.837	-47.827	-57.012	-52.100	-68.340	-75.268	-71.661
India	1.758 (1.654-1.873)	2.120 (2.003-2.241)	1.931 (1.820-2.045)	1.141 (1.054-1.231)	1.225 (1.146-1.306)	1.181 (1.100-1.264)	0.561 (0.501-0.632)	0.595 (0.538-0.662)	0.577 (0.522-0.642)	-35.106	-42.203	-38.856	-50.864	-51.434	-51.135	-68.114	-71.930	-70.122
Indonesia	1.314 (1.218-1.403)	1.126 (1.022-1.208)	1.222 (1.127-1.304)	0.779 (0.674-0.870)	0.618 (0.505-0.719)	0.701 (0.594-0.793)	0.481 (0.420-0.541)	0.324 (0.283-0.369)	0.405 (0.358-0.452)	-40.713	-45.076	-42.656	-38.253	-47.647	-42.274	-63.392	-71.246	-66.897
Kenya	1.016 (0.853-1.174)	0.811 (0.630-0.992)	0.914 (0.743-1.083)	1.304 (1.183-1.482)	1.047 (0.923-1.228)	1.177 (1.057-1.357)	0.726 (0.626-0.852)	0.533 (0.443-0.672)	0.631 (0.540-0.756)	28.321	29.066	28.774	-44.332	-49.067	-46.384	-28.566	-34.262	-30.956
Kiribati	1.371 (1.156-1.590)	0.990 (0.878-1.106)	1.186 (1.022-1.353)	1.085 (0.919-1.263)	0.774 (0.691-0.857)	0.934 (0.812-1.059)	0.788 (0.691-0.897)	0.570 (0.502-0.643)	0.682 (0.599-0.773)	-20.854	-21.868	-21.222	-27.350	-26.365	-27.030	-42.500	-42.467	-42.515
Kyrgyzstan	0.848 (0.798-0.905)	0.536 (0.509-0.565)	0.693 (0.656-0.736)	0.400 (0.383-0.418)	0.266 (0.256-0.276)	0.334 (0.321-0.349)	0.311 (0.293-0.331)	0.199 (0.188-0.211)	0.256 (0.242-0.272)	-52.852	-50.458	-51.856	-22.114	-25.176	-23.217	-63.278	-62.931	-63.034
Lao People's Democratic Republic	2.442 (2.078-2.787)	1.998 (1.740-2.235)	2.223 (1.911-2.519)	1.379 (1.183-1.540)	1.078 (0.934-1.184)	1.231 (1.059-1.365)	0.650 (0.506-0.774)	0.470 (0.364-0.550)	0.562 (0.437-0.662)	-43.530	-46.059	-44.641	-52.832	-56.385	-54.346	-73.364	-76.474	-74.727
Lesotho	1.395 (1.050-1.616)	0.950 (0.681-1.085)	1.173 (0.866-1.351)	1.885 (1.739-2.054)	1.161 (1.053-1.279)	1.524 (1.400-1.664)	1.610 (1.443-1.791)	1.086 (0.964-1.210)	1.349 (1.206-1.493)	35.061	22.257	29.931	-14.551	-6.481	-11.503	15.409	14.334	14.985
Mauritania	1.562 (1.410-1.701)	1.336 (1.237-1.429)	1.451 (1.323-1.564)	1.056 (0.850-1.191)	0.938 (0.704-1.049)	0.998 (0.776-1.121)	0.680 (0.486-0.940)	0.595 (0.385-0.827)	0.638 (0.436-0.882)	-32.380	-29.783	-31.225	-35.622	-36.593	-36.085	-56.468	-55.478	-56.043
Micronesia (Federated States of)	0.882 (0.759-1.003)	0.610 (0.528-0.692)	0.751 (0.647-0.852)	0.610 (0.534-0.696)	0.437 (0.389-0.493)	0.526 (0.463-0.596)	0.460 (0.398-0.523)	0.345 (0.300-0.391)	0.404 (0.350-0.459)	-30.805	-28.432	-29.955	-24.682	-20.982	-23.248	-47.884	-43.448	-46.239
Mongolia	0.949 (0.886-1.014)	0.764 (0.719-0.810)	0.857 (0.804-0.911)	0.715 (0.652-0.787)	0.478 (0.448-0.511)	0.598 (0.553-0.649)	0.460 (0.388-0.554)	0.299 (0.262-0.347)	0.381 (0.326-0.452)	-24.604	-37.365	-30.217	-35.700	-37.409	-36.275	-51.521	-60.796	-55.531
Morocco	0.837 (0.717-1.000)	0.684 (0.559-0.876)	0.761 (0.641-0.936)	0.837 (0.739-0.938)	0.461 (0.379-0.600)	0.652 (0.566-0.754)	0.335 (0.263-0.404)	0.255 (0.221-0.291)	0.296 (0.243-0.348)	-0.005	-32.522	-14.318	-59.971	-44.812	-54.622	-59.973	-62.760	-61.119
Myanmar	2.184 (1.834-2.485)	1.694 (1.435-1.882)	1.942 (1.639-2.186)	1.401 (1.171-1.582)	1.064 (0.900-1.185)	1.233 (1.036-1.384)	0.641 (0.527-0.820)	0.427 (0.340-0.556)	0.536 (0.435-0.690)	-35.866	-37.190	-36.479	-54.242	-59.837	-56.572	-70.654	-74.774	-72.414
Nicaragua	0.680 (0.643-0.714)	0.491 (0.469-0.514)	0.586 (0.558-0.615)	0.451 (0.421-0.478)	0.287 (0.270-0.304)	0.371 (0.347-0.392)	0.309 (0.267-0.356)	0.209 (0.183-0.239)	0.260 (0.227-0.299)	-33.630	-41.542	-36.766	-31.447	-27.110	-29.761	-54.501	-57.390	-55.585
Nigeria	1.769 (1.549-2.040)	1.365 (1.106-1.689)	1.557 (1.329-1.844)	1.484 (1.288-1.761)	1.155 (0.942-1.519)	1.323 (1.129-1.638)	0.989 (0.863-1.258)	0.739 (0.635-1.113)	0.862 (0.755-1.193)	-16.110	-15.384	-14.976	-33.361	-36.028	-34.857	-44.096	-45.870	-44.613
Pakistan	1.306 (1.216-1.392)	1.199 (1.099-1.292)	1.255 (1.166-1.341)	1.703 (1.582-1.827)	1.483 (1.387-1.569)	1.597 (1.490-1.698)	1.018 (0.801-1.191)	0.919 (0.660-1.079)	0.970 (0.745-1.127)	30.365	23.687	27.261	-40.203	-38.045	-39.243	-22.046	-23.370	-22.680
Palestine	0.749 (0.698-0.806)	0.571 (0.536-0.614)	0.662 (0.619-0.713)	0.748 (0.705-0.793)	0.660 (0.623-0.700)	0.705 (0.666-0.745)	0.295 (0.265-0.331)	0.202 (0.184-0.224)	0.250 (0.225-0.279)	-0.108	15.630	6.404	-60.519	-69.307	-64.526	-60.562	-64.510	-62.255

Papua New Guinea	1.347 (1.102-1.540)	1.133 (0.949-1.257)	1.245 (1.032-1.403)	1.146 (0.949-1.293)	0.946 (0.800-1.068)	1.051 (0.884-1.185)	0.912 (0.760-1.027)	0.728 (0.636-0.768)	0.824 (0.705-0.900)	-14.941	-16.488	-15.556	-20.418	-23.059	-21.598	-32.308	-35.745	-33.794
Philippines	1.367 (1.278-1.457)	1.082 (1.023-1.141)	1.227 (1.156-1.301)	0.814 (0.751-0.879)	0.634 (0.598-0.672)	0.726 (0.679-0.777)	0.642 (0.565-0.724)	0.521 (0.469-0.576)	0.583 (0.526-0.649)	-40.494	-41.421	-40.853	-21.119	-17.804	-19.654	-53.061	-51.850	-52.478
Republic of Moldova	0.727 (0.679-0.783)	0.438 (0.412-0.467)	0.585 (0.546-0.627)	0.509 (0.469-0.551)	0.310 (0.291-0.329)	0.411 (0.382-0.442)	0.268 (0.240-0.301)	0.200 (0.181-0.221)	0.235 (0.211-0.262)	-30.017	-29.292	-29.654	-47.394	-35.476	-42.959	-63.185	-54.376	-59.874
Sao Tome and Principe	2.075 (1.941-2.207)	0.889 (0.726-1.084)	1.488 (1.350-1.638)	0.905 (0.790-1.007)	0.814 (0.711-0.898)	0.860 (0.752-0.952)	0.467 (0.388-0.551)	0.354 (0.305-0.410)	0.411 (0.346-0.481)	-56.403	-8.423	-42.190	-48.377	-56.517	-52.256	-77.494	-60.180	-72.399
Senegal	1.545 (1.387-1.684)	1.252 (1.091-1.381)	1.397 (1.236-1.523)	1.117 (0.920-1.274)	0.923 (0.709-1.072)	1.021 (0.813-1.171)	0.769 (0.594-0.985)	0.638 (0.450-0.828)	0.706 (0.525-0.911)	-27.710	-26.284	-26.876	-31.184	-30.871	-30.875	-50.253	-49.041	-49.453
Solomon Islands	1.065 (0.922-1.217)	0.793 (0.696-0.906)	0.935 (0.813-1.065)	0.901 (0.790-1.012)	0.681 (0.612-0.754)	0.796 (0.703-0.888)	0.671 (0.584-0.761)	0.518 (0.453-0.585)	0.598 (0.520-0.677)	-15.448	-14.133	-14.860	-25.453	-23.901	-24.839	-36.969	-34.655	-36.008
Sudan	1.553 (1.342-1.781)	1.261 (1.050-1.492)	1.411 (1.200-1.642)	0.963 (0.792-1.284)	0.749 (0.582-1.033)	0.860 (0.692-1.161)	0.606 (0.513-0.783)	0.450 (0.363-0.627)	0.531 (0.44-0.708)	-38.003	-40.643	-39.081	-37.061	-39.855	-38.277	-60.980	-64.299	-62.399
Timor-Leste	1.564 (1.180-1.845)	1.440 (1.016-1.729)	1.506 (1.101-1.786)	0.906 (0.701-1.045)	0.775 (0.570-0.896)	0.843 (0.639-0.972)	0.531 (0.459-0.618)	0.390 (0.334-0.455)	0.463 (0.399-0.539)	-42.073	-46.163	-44.051	-41.379	-49.653	-45.034	-66.042	-72.895	-69.247
Tunisia	0.794 (0.736-0.856)	0.609 (0.571-0.650)	0.703 (0.655-0.756)	0.449 (0.384-0.522)	0.343 (0.302-0.387)	0.397 (0.345-0.456)	0.282 (0.235-0.339)	0.215 (0.184-0.253)	0.250 (0.211-0.297)	-43.452	-43.703	-43.522	-37.085	-37.146	-37.002	-64.423	-64.615	-64.420
Ukraine	0.580 (0.532-0.629)	0.326 (0.304-0.348)	0.455 (0.420-0.491)	0.362 (0.357-0.367)	0.219 (0.216-0.222)	0.292 (0.289-0.296)	0.328 (0.297-0.358)	0.219 (0.200-0.238)	0.275 (0.254-0.296)	-37.601	-32.685	-35.720	-9.431	-0.238	-6.009	-43.486	-32.846	-39.583
Uzbekistan	0.749 (0.699-0.798)	0.484 (0.454-0.515)	0.618 (0.578-0.657)	0.735 (0.667-0.806)	0.519 (0.484-0.559)	0.629 (0.577-0.686)	0.516 (0.458-0.586)	0.375 (0.339-0.418)	0.448 (0.400-0.505)	-1.879	7.347	1.823	-29.807	-27.762	-28.831	-31.126	-22.454	-27.534
Vanuatu	0.779 (0.643-0.912)	0.545 (0.464-0.622)	0.667 (0.558-0.773)	0.745 (0.619-0.867)	0.524 (0.449-0.597)	0.639 (0.538-0.739)	0.592 (0.507-0.678)	0.422 (0.364-0.476)	0.510 (0.438-0.581)	-4.345	-3.725	-4.159	-20.565	-19.551	-20.233	-24.016	-22.548	-23.550
Viet Nam	0.683 (0.606-0.855)	0.420 (0.359-0.548)	0.554 (0.486-0.705)	0.459 (0.409-0.544)	0.264 (0.232-0.316)	0.364 (0.325-0.432)	0.326 (0.286-0.371)	0.195 (0.172-0.220)	0.263 (0.232-0.297)	-32.738	-37.211	-34.308	-29.122	-25.902	-27.776	-52.326	-53.475	-52.554
Zambia	1.927 (1.745-2.119)	1.600 (1.464-1.733)	1.762 (1.605-1.919)	2.109 (1.918-2.326)	1.679 (1.502-1.848)	1.893 (1.711-2.081)	0.985 (0.805-1.202)	0.722 (0.582-0.912)	0.853 (0.696-1.058)	9.495	4.972	7.464	-53.283	-57.030	-54.940	-48.847	-54.894	-51.577
Zimbabwe	1.004 (0.872-1.104)	0.731 (0.604-0.808)	0.867 (0.738-0.953)	1.825 (1.568-2.076)	1.340 (1.087-1.578)	1.582 (1.327-1.823)	1.211 (1.080-1.375)	1.010 (0.922-1.124)	1.110 (1.002-1.248)	81.838	83.203	82.485	-33.676	-24.583	-29.798	20.602	38.166	28.107
**59 Upper MICs**																		
Albania	0.642 (0.599-0.689)	0.506 (0.480-0.535)	0.577 (0.543-0.614)	0.659 (0.601-0.722)	0.460 (0.431-0.492)	0.562 (0.519-0.609)	0.416 (0.367-0.476)	0.268 (0.241-0.300)	0.346 (0.307-0.393)	2.572	-8.941	-2.613	-36.769	-41.870	-38.412	-35.142	-47.067	-40.022
Algeria	1.285 (1.192-1.390)	0.956 (0.888-1.028)	1.124 (1.045-1.212)	0.636 (0.586-0.687)	0.498 (0.462-0.536)	0.568 (0.526-0.612)	0.364 (0.326-0.405)	0.311 (0.285-0.336)	0.338 (0.306-0.371)	-50.554	-47.883	-49.455	-42.687	-37.663	-40.511	-71.661	-67.512	-69.931
American Samoa	0.400 (0.353-0.457)	0.283 (0.255-0.318)	0.345 (0.307-0.391)	0.327 (0.297-0.368)	0.240 (0.224-0.267)	0.285 (0.262-0.318)	0.266 (0.224-0.333)	0.213 (0.178-0.265)	0.241 (0.202-0.301)	-18.100	-15.148	-17.171	-18.683	-11.382	-15.646	-33.401	-24.806	-30.131
Argentina	0.399 (0.396-0.402)	0.268 (0.266-0.269)	0.334 (0.332-0.336)	0.317 (0.314-0.321)	0.220 (0.218-0.222)	0.269 (0.267-0.271)	0.229 (0.222-0.236)	0.173 (0.171-0.175)	0.201 (0.196-0.206)	-20.637	-17.916	-19.463	-27.779	-21.490	-25.228	-42.683	-35.556	-39.780
Armenia	0.535 (0.505-0.568)	0.327 (0.310-0.347)	0.434 (0.410-0.459)	0.297 (0.274-0.320)	0.197 (0.185-0.21)	0.249 (0.231-0.267)	0.238 (0.211-0.269)	0.168 (0.153-0.185)	0.205 (0.184-0.230)	-44.437	-39.752	-42.538	-20.028	-14.847	-17.681	-55.565	-48.697	-52.699
Azerbaijan	0.834 (0.774-0.902)	0.588 (0.550-0.630)	0.714 (0.666-0.769)	0.829 (0.763-0.902)	0.645 (0.603-0.693)	0.741 (0.686-0.802)	0.450 (0.387-0.529)	0.353 (0.319-0.393)	0.405 (0.356-0.466)	-0.551	9.772	3.747	-45.776	-45.332	-45.371	-46.075	-39.990	-43.324
Belarus	0.527 (0.478-0.580)	0.312 (0.287-0.339)	0.421 (0.384-0.461)	0.335 (0.302-0.370)	0.188 (0.173-0.204)	0.264 (0.239-0.289)	0.190 (0.160-0.230)	0.137 (0.118-0.162)	0.164 (0.140-0.197)	-36.315	-39.770	-37.413	-43.358	-27.010	-37.679	-63.928	-56.037	-60.995
Belize	0.630 (0.586-0.677)	0.442 (0.412-0.470)	0.537 (0.501-0.574)	0.515 (0.477-0.558)	0.39 (0.366-0.419)	0.453 (0.423-0.487)	0.343 (0.309-0.383)	0.227 (0.208-0.251)	0.286 (0.259-0.317)	-18.297	-11.704	-15.586	-33.321	-41.746	-36.939	-45.521	-48.564	-46.768
Bosnia and Herzegovina	0.334	0.189	0.263	0.172	0.117	0.146	0.140	0.101	0.121	-48.442	-37.693	-44.643	-18.647	-13.771	-16.738	-58.056	-46.273	-53.909

	(0.326-0.344)	(0.184-0.193)	(0.257-0.270)	(0.166-0.179)	(0.113-0.122)	(0.141-0.151)	(0.118-0.166)	(0.088-0.117)	(0.103-0.143)									
Botswana	0.994	0.660	0.824	1.445	0.959	1.203	0.779	0.535	0.658	45.471	45.296	45.883	-46.119	-44.242	-45.275	-21.619	-18.986	-20.166
	(0.799-1.140)	(0.541-0.766)	(0.668-0.934)	(1.166-1.616)	(0.775-1.082)	(0.972-1.349)	(0.612-0.942)	(0.416-0.624)	(0.515-0.784)									
Brazil	0.709	0.457	0.584	0.508	0.348	0.429	0.352	0.238	0.296	-28.358	-23.766	-26.512	-30.692	-31.727	-31.073	-50.346	-47.953	-49.347
	(0.679-0.743)	(0.440-0.474)	(0.561-0.609)	(0.476-0.541)	(0.331-0.366)	(0.405-0.454)	(0.318-0.391)	(0.220-0.257)	(0.270-0.324)									
Bulgaria	0.529	0.347	0.440	0.365	0.274	0.321	0.222	0.159	0.191	-30.912	-21.089	-27.130	-39.327	-41.786	-40.324	-58.082	-54.063	-56.515
	(0.514-0.544)	(0.338-0.355)	(0.430-0.451)	(0.352-0.380)	(0.266-0.282)	(0.311-0.331)	(0.187-0.262)	(0.139-0.183)	(0.164-0.224)									
China	0.902	0.553	0.734	0.535	0.299	0.425	0.291	0.169	0.235	-40.633	-45.950	-42.030	-45.568	-43.322	-44.639	-67.686	-69.366	-67.907
	(0.833-0.97)	(0.506-0.631)	(0.680-0.795)	(0.502-0.569)	(0.283-0.316)	(0.401-0.450)	(0.263-0.323)	(0.156-0.183)	(0.215-0.257)									
Colombia	0.759	0.497	0.629	0.539	0.322	0.433	0.345	0.243	0.295	-28.972	-35.215	-31.223	-36.051	-24.453	-31.819	-54.578	-51.057	-53.108
	(0.715-0.801)	(0.472-0.521)	(0.595-0.662)	(0.501-0.578)	(0.302-0.340)	(0.404-0.462)	(0.291-0.413)	(0.212-0.280)	(0.252-0.348)									
Costa Rica	0.323	0.244	0.284	0.263	0.181	0.223	0.212	0.145	0.179	-18.432	-25.650	-21.537	-19.650	-20.140	-19.854	-34.460	-40.624	-37.116
	(0.315-0.331)	(0.238-0.250)	(0.278-0.291)	(0.256-0.272)	(0.176-0.186)	(0.217-0.229)	(0.179-0.250)	(0.126-0.167)	(0.153-0.209)									
Cuba	0.435	0.320	0.379	0.273	0.161	0.219	0.198	0.136	0.168	-37.173	-49.651	-42.259	-27.337	-15.432	-23.061	-54.348	-57.420	-55.575
	(0.426-0.443)	(0.314-0.326)	(0.372-0.386)	(0.266-0.281)	(0.157-0.165)	(0.213-0.224)	(0.172-0.228)	(0.121-0.153)	(0.147-0.192)									
Dominica	0.433	0.365	0.400	0.547	0.373	0.461	0.462	0.398	0.430	26.404	2.153	15.130	-15.689	6.726	-6.670	6.572	9.024	7.451
	(0.382-0.482)	(0.330-0.398)	(0.357-0.440)	(0.491-0.606)	(0.329-0.418)	(0.413-0.510)	(0.388-0.550)	(0.333-0.468)	(0.361-0.510)									
Dominican Republic	0.754	0.537	0.645	0.594	0.387	0.492	0.465	0.306	0.387	-21.176	-27.977	-23.729	-21.848	-20.818	-21.377	-38.398	-42.970	-40.034
	(0.704-0.810)	(0.505-0.573)	(0.605-0.690)	(0.541-0.649)	(0.358-0.418)	(0.451-0.534)	(0.387-0.555)	(0.259-0.359)	(0.324-0.459)									
Ecuador	0.872	0.649	0.762	0.764	0.557	0.662	0.431	0.345	0.389	-12.398	-14.129	-13.095	-43.579	-38.187	-41.309	-50.573	-46.921	-48.994
	(0.824-0.922)	(0.615-0.681)	(0.720-0.803)	(0.697-0.837)	(0.509-0.606)	(0.606-0.720)	(0.379-0.487)	(0.310-0.381)	(0.345-0.436)									
Equatorial Guinea	2.408	1.776	2.099	1.205	0.946	1.090	0.810	0.618	0.726	-49.940	-46.718	-48.061	-32.833	-34.693	-33.380	-66.376	-65.203	-65.398
	(2.043-2.721)	(1.534-1.976)	(1.821-2.351)	(0.877-1.642)	(0.631-1.297)	(0.765-1.487)	(0.588-1.037)	(0.422-0.791)	(0.516-0.933)									
Fiji	0.679	0.604	0.642	0.698	0.492	0.598	0.606	0.450	0.530	2.921	-18.574	-6.824	-13.223	-8.519	-11.470	-10.688	-25.511	-17.511
	(0.585-0.792)	(0.536-0.687)	(0.561-0.741)	(0.626-0.773)	(0.449-0.535)	(0.541-0.657)	(0.508-0.725)	(0.389-0.522)	(0.450-0.626)									
Gabon	1.378	0.966	1.170	1.044	0.724	0.880	0.638	0.395	0.514	-24.265	-25.042	-24.780	-38.867	-45.518	-41.619	-53.701	-59.162	-56.086
	(1.249-1.499)	(0.861-1.061)	(1.059-1.273)	(0.910-1.221)	(0.615-0.878)	(0.759-1.042)	(0.550-0.739)	(0.333-0.455)	(0.440-0.594)									
Georgia	0.634	0.395	0.517	0.304	0.218	0.263	0.240	0.161	0.202	-51.970	-44.840	-49.174	-21.238	-26.173	-22.926	-62.171	-59.277	-60.826
	(0.587-0.683)	(0.370-0.423)	(0.480-0.555)	(0.277-0.334)	(0.203-0.234)	(0.241-0.286)	(0.213-0.272)	(0.145-0.180)	(0.181-0.228)									
Grenada	0.421	0.496	0.458	0.455	0.286	0.369	0.301	0.226	0.265	8.002	-42.306	-19.407	-33.810	-20.902	-28.228	-28.513	-54.365	-42.157
	(0.370-0.480)	(0.445-0.555)	(0.407-0.516)	(0.408-0.504)	(0.263-0.311)	(0.334-0.406)	(0.264-0.340)	(0.204-0.250)	(0.235-0.296)									
Guatemala	1.834	1.612	1.724	0.689	0.557	0.624	0.474	0.377	0.426	-62.457	-65.485	-63.790	-31.211	-32.266	-31.716	-74.175	-76.622	-75.274
	(1.700-1.970)	(1.485-1.737)	(1.601-1.848)	(0.634-0.749)	(0.516-0.596)	(0.577-0.672)	(0.398-0.565)	(0.326-0.437)	(0.363-0.502)									
Guyana	0.934	0.734	0.833	0.738	0.555	0.648	0.577	0.423	0.501	-20.971	-24.457	-22.246	-21.830	-23.683	-22.668	-38.223	-42.348	-39.871
	(0.856-1.017)	(0.684-0.786)	(0.772-0.900)	(0.668-0.815)	(0.515-0.597)	(0.595-0.705)	(0.482-0.691)	(0.365-0.492)	(0.424-0.593)									
Iran (Islamic Republic of)	1.514	1.073	1.298	0.822	0.567	0.697	0.372	0.284	0.329	-45.719	-47.199	-46.287	-54.790	-49.825	-52.814	-75.460	-73.507	-74.655
	(1.428-1.609)	(1.018-1.131)	(1.228-1.374)	(0.758-0.882)	(0.530-0.600)	(0.648-0.744)	(0.353-0.389)	(0.271-0.296)	(0.314-0.344)									
Iraq	1.422	1.008	1.220	1.088	0.821	0.957	0.459	0.348	0.405	-23.507	-18.587	-21.515	-57.828	-57.611	-57.728	-67.741	-65.489	-66.823
	(1.320-1.520)	(0.954-1.063)	(1.145-1.295)	(0.990-1.189)	(0.751-0.885)	(0.876-1.038)	(0.402-0.527)	(0.31-0.398)	(0.356-0.465)									
Jamaica	0.406	0.352	0.379	0.247	0.215	0.231	0.240	0.186	0.214	-39.120	-38.929	-38.984	-2.935	-13.464	-7.752	-40.907	-47.152	-43.714
	(0.375-0.439)	(0.328-0.377)	(0.351-0.408)	(0.223-0.276)	(0.198-0.234)	(0.211-0.255)	(0.201-0.287)	(0.161-0.216)	(0.181-0.252)									
Jordan	0.614	0.526	0.571	0.536	0.442	0.490	0.317	0.239	0.279	-12.718	-16.026	-14.200	-40.931	-46.001	-43.152	-48.444	-54.655	-51.224
	(0.573-0.661)	(0.492-0.561)	(0.536-0.610)	(0.493-0.586)	(0.410-0.476)	(0.454-0.530)	(0.282-0.360)	(0.211-0.274)	(0.248-0.317)									
Kazakhstan	0.795	0.430	0.614	0.688	0.416	0.554	0.361	0.235	0.299	-13.501	-3.243	-9.767	-47.501	-43.553	-46.010	-54.589	-45.383	-51.283
	(0.743-0.851)	(0.406-0.456)	(0.577-0.655)	(0.632-0.748)	(0.386-0.447)	(0.513-0.601)	(0.321-0.408)	(0.212-0.261)	(0.268-0.337)									
Lebanon	0.692	0.565	0.632	0.292	0.229	0.262	0.212	0.163	0.188	-57.846	-59.531	-58.540	-27.430	-28.876	-28.114	-69.409	-71.217	-70.196
	(0.622-0.780)	(0.497-0.641)	(0.563-0.714)	(0.245-0.334)	(0.197-0.257)	(0.223-0.297)	(0.178-0.247)	(0.143-0.187)	(0.162-0.218)									
Libya	0.688	0.502	0.594	0.319	0.282	0.300	0.376	0.336	0.356	-53.675	-43.906	-49.394	17.925	19.164	18.540	-45.371	-33.156	-40.012

	(0.613-0.771)	(0.453-0.560)	(0.534-0.662)	(0.287-0.351)	(0.258-0.307)	(0.274-0.329)	(0.341-0.412)	(0.310-0.364)	(0.326-0.388)									
Malaysia	0.562	0.401	0.483	0.340	0.238	0.291	0.282	0.195	0.240	-39.484	-40.561	-39.852	-17.207	-18.094	-17.573	-49.897	-51.316	-50.422
	(0.554-0.570)	(0.395-0.407)	(0.478-0.489)	(0.334-0.346)	(0.235-0.242)	(0.287-0.295)	(0.238-0.333)	(0.171-0.224)	(0.206-0.280)									
Maldives	0.921	1.011	0.965	0.364	0.266	0.316	0.268	0.193	0.232	-60.481	-73.681	-67.208	-26.363	-27.551	-26.805	-70.900	-80.932	-75.998
	(0.852-0.992)	(0.947-1.077)	(0.900-1.034)	(0.337-0.392)	(0.247-0.286)	(0.294-0.340)	(0.238-0.305)	(0.175-0.213)	(0.208-0.261)									
Marshall Islands	0.632	0.513	0.574	0.719	0.526	0.625	0.509	0.377	0.444	13.767	2.650	8.875	-29.141	-28.370	-28.869	-19.387	-26.472	-22.556
	(0.590-0.674)	(0.481-0.546)	(0.539-0.611)	(0.640-0.789)	(0.470-0.576)	(0.557-0.685)	(0.416-0.624)	(0.310-0.452)	(0.363-0.539)									
Mauritius	0.367	0.294	0.331	0.227	0.213	0.220	0.194	0.160	0.177	-38.161	-27.523	-33.497	-14.564	-24.996	-19.561	-47.167	-45.639	-46.506
	(0.354-0.380)	(0.284-0.304)	(0.320-0.342)	(0.218-0.237)	(0.205-0.222)	(0.211-0.229)	(0.167-0.225)	(0.140-0.183)	(0.154-0.204)									
Mexico	0.687	0.500	0.594	0.397	0.292	0.345	0.300	0.231	0.266	-42.181	-41.589	-41.897	-24.545	-21.020	-23.005	-56.373	-53.867	-55.264
	(0.650-0.729)	(0.474-0.526)	(0.563-0.627)	(0.370-0.427)	(0.278-0.307)	(0.324-0.367)	(0.266-0.339)	(0.210-0.253)	(0.240-0.295)									
Montenegro	0.279	0.191	0.236	0.186	0.152	0.170	0.141	0.084	0.114	-33.238	-20.501	-28.161	-24.400	-44.538	-32.954	-49.528	-55.908	-51.835
	(0.262-0.297)	(0.182-0.202)	(0.223-0.250)	(0.177-0.197)	(0.146-0.159)	(0.162-0.178)	(0.121-0.165)	(0.075-0.095)	(0.099-0.132)									
Namibia	1.146	0.825	0.985	1.263	0.807	1.033	0.843	0.539	0.690	10.201	-2.263	4.830	-33.316	-33.146	-33.171	-26.513	-34.659	-29.943
	(1.023-1.276)	(0.698-0.935)	(0.862-1.104)	(1.153-1.375)	(0.728-0.881)	(0.940-1.124)	(0.660-1.018)	(0.424-0.633)	(0.543-0.824)									
Nauru	0.751	0.532	0.644	0.986	0.679	0.838	0.611	0.436	0.527	31.276	27.615	30.184	-38.045	-35.831	-37.176	-18.668	-18.111	-18.214
	(0.669-0.837)	(0.482-0.585)	(0.578-0.711)	(0.883-1.093)	(0.620-0.739)	(0.758-0.921)	(0.539-0.707)	(0.379-0.509)	(0.462-0.608)									
North Macedonia	0.379	0.258	0.321	0.213	0.127	0.171	0.184	0.121	0.154	-43.931	-50.887	-46.613	-13.348	-4.310	-10.058	-51.415	-53.004	-51.983
	(0.369-0.391)	(0.252-0.266)	(0.312-0.329)	(0.205-0.221)	(0.122-0.132)	(0.165-0.178)	(0.156-0.218)	(0.106-0.140)	(0.132-0.180)									
Paraguay	0.486	0.396	0.442	0.454	0.332	0.394	0.287	0.238	0.263	-6.490	-16.093	-10.676	-36.705	-28.430	-33.247	-40.813	-39.948	-40.374
	(0.448-0.524)	(0.367-0.424)	(0.410-0.475)	(0.417-0.494)	(0.309-0.357)	(0.365-0.426)	(0.240-0.344)	(0.206-0.275)	(0.223-0.310)									
Peru	1.001	0.878	0.940	0.566	0.436	0.502	0.353	0.302	0.329	-43.490	-50.292	-46.592	-37.568	-30.683	-34.507	-64.720	-65.544	-65.022
	(0.939-1.068)	(0.831-0.926)	(0.886-0.997)	(0.522-0.612)	(0.407-0.467)	(0.466-0.539)	(0.295-0.424)	(0.261-0.352)	(0.278-0.389)									
Romania	0.631	0.399	0.518	0.368	0.256	0.314	0.216	0.151	0.184	-41.633	-35.825	-39.425	-41.494	-41.076	-41.294	-65.852	-62.186	-64.439
	(0.623-0.639)	(0.394-0.404)	(0.513-0.523)	(0.363-0.375)	(0.251-0.261)	(0.309-0.319)	(0.195-0.241)	(0.139-0.165)	(0.168-0.204)									
Russian Federation	0.639	0.334	0.489	0.510	0.305	0.409	0.244	0.172	0.209	-20.221	-8.845	-16.234	-52.166	-43.629	-49.046	-61.838	-48.615	-57.318
	(0.635-0.643)	(0.332-0.336)	(0.486-0.491)	(0.505-0.514)	(0.302-0.307)	(0.406-0.412)	(0.221-0.267)	(0.159-0.186)	(0.192-0.226)									
Saint Lucia	0.446	0.267	0.356	0.440	0.297	0.370	0.307	0.258	0.283	-0.986	11.256	3.762	-30.432	-13.176	-23.487	-31.118	-3.403	-20.608
	(0.401-0.492)	(0.244-0.291)	(0.324-0.391)	1(0.396-0.49)	(0.270-0.325)	(0.334-0.407)	(0.263-0.361)	(0.227-0.293)	(0.245-0.327)									
Saint Vincent and the Grenadines	0.534	0.451	0.493	0.412	0.471	0.441	0.308	0.300	0.304	-22.775	4.278	-10.544	-25.254	-36.192	-31.002	-42.278	-33.462	-38.277
	(0.475-0.592)	(0.410-0.494)	(0.443-0.542)	(0.374-0.455)	(0.439-0.508)	(0.407-0.479)	(0.266-0.354)	(0.268-0.336)	(0.267-0.345)									
Samoa	0.698	0.485	0.599	0.500	0.347	0.427	0.342	0.246	0.296	-28.375	-28.525	-28.654	-31.631	-29.108	-30.789	-51.031	-49.330	-50.621
	(0.579-0.867)	(0.416-0.574)	(0.502-0.73)	(0.421-0.606)	(0.293-0.423)	(0.360-0.518)	(0.267-0.432)	(0.194-0.311)	(0.232-0.374)									
Serbia	0.445	0.289	0.367	0.230	0.150	0.191	0.098	0.073	0.086	-48.281	-48.033	-47.852	-57.399	-51.315	-54.787	-77.967	-74.700	-76.423
	(0.436-0.454)	(0.284-0.294)	(0.360-0.373)	(0.210-0.251)	(0.140-0.161)	(0.176-0.207)	(0.087-0.110)	(0.066-0.081)	(0.077-0.097)									
South Africa	1.099	0.834	0.966	1.346	1.097	1.222	0.803	0.629	0.717	22.393	31.449	26.457	-40.341	-42.665	-41.332	-26.981	-24.634	-25.811
	(1.046-1.157)	(0.799-0.869)	(0.923-1.01)	(1.242-1.458)	(1.032-1.165)	(1.138-1.312)	(0.724-0.897)	(0.580-0.688)	(0.653-0.793)									
Sri Lanka	0.885	0.803	0.845	0.541	0.499	0.520	0.264	0.229	0.247	-38.930	-37.905	-38.459	-51.116	-54.012	-52.476	-70.147	-71.443	-70.753
	(0.848-0.924)	(0.780-0.829)	(0.816-0.875)	(0.525-0.556)	(0.485-0.513)	(0.506-0.534)	(0.221-0.317)	(0.198-0.266)	(0.209-0.292)									
Suriname	0.647	0.647	0.647	0.534	0.494	0.514	0.407	0.365	0.387	-17.488	-23.643	-20.505	-23.642	-26.227	-24.805	-36.995	-43.669	-40.224
	(0.593-0.698)	(0.605-0.688)	(0.599-0.692)	(0.486-0.583)	(0.454-0.538)	(0.471-0.561)	(0.348-0.477)	(0.323-0.412)	(0.336-0.446)									
Thailand	1.085	0.800	0.945	0.651	0.446	0.552	0.413	0.270	0.343	-39.943	-44.204	-41.605	-36.562	-39.572	-37.760	-61.901	-66.283	-63.655
	(0.992-1.194)	(0.741-0.873)	(0.870-1.037)	(0.602-0.710)	(0.416-0.480)	(0.512-0.598)	(0.372-0.458)	(0.247-0.294)	(0.311-0.378)									
Tonga	0.515	0.311	0.417	0.441	0.253	0.352	0.338	0.206	0.275	-14.218	-18.541	-15.531	-23.420	-18.653	-21.901	-34.308	-33.735	-34.030
	(0.458-0.576)	(0.282-0.344)	(0.374-0.464)	(0.387-0.498)	(0.226-0.283)	(0.312-0.396)	(0.284-0.405)	(0.184-0.249)	(0.236-0.330)									
Turkey	0.732	0.520	0.629	0.348	0.280	0.315	0.256	0.197	0.227	-52.402	-46.265	-49.946	-26.597	-29.529	-27.857	-65.062	-62.132	-63.890
	(0.679-0.786)	(0.494-0.548)	(0.589-0.670)	(0.319-0.380)	(0.258-0.301)	(0.290-0.341)	(0.228-0.287)	(0.179-0.219)	(0.204-0.254)									
Turkmenistan	0.782	0.533	0.659	0.649	0.456	0.556	0.405	0.290	0.350	-17.051	-14.525	-15.694	-37.609	-36.258	-37.068	-48.247	-45.516	-46.944

	(0.717-0.856)	(0.501-0.568)	(0.611-0.713)	(0.603-0.701)	(0.427-0.487)	(0.518-0.597)	(0.359-0.460)	(0.264-0.323)	(0.313-0.393)									
Tuvalu	1.107 (0.969-1.248)	0.862 (0.753-1.032)	0.991 (0.871-1.143)	0.623 (0.536-0.709)	0.456 (0.392-0.514)	0.544 (0.467-0.616)	0.389 (0.310-0.489)	0.299 (0.237-0.370)	0.347 (0.275-0.433)	-43.704	-47.141	-45.121	-37.508	-34.398	-36.262	-64.820	-65.323	-65.021
Venezuela (Bolivarian Republic of)	0.567 (0.560-0.574)	0.391 (0.386-0.396)	0.480 (0.476-0.484)	0.399 (0.393-0.404)	0.281 (0.277-0.285)	0.341 (0.338-0.345)	0.365 (0.303-0.437)	0.241 (0.206-0.280)	0.304 (0.256-0.361)	-29.693	-28.143	-28.981	-8.602	-14.185	-10.833	-35.741	-38.336	-36.674

Schedule2 Sex-causes mortality and its rank in children age 5-14 years in region-specific from 1990 to 2019

	global						LICs						Lower MICs						Upper MICs					
	boy		girl		total		boy		girl		total		boy		girl		total		boy		girl		total	
cause of death	rate	rank	rate	rank	rate	Rank	rate	rank	rate	rank	rate	rank	rate	rank	rate	rank	rate	rank	rate	rank	rate	rank	rate	rank
1990 year																								
Cardiovascular diseases	0.024 (0.021-0.026)	12	0.025 (0.022-0.028)	11	0.024 (0.022-0.027)	12	0.034 (0.027-0.042)	12	0.028 (0.022-0.034)	12	0.031 (0.025-0.037)	12	0.025 (0.022-0.029)	12	0.034 (0.028-0.040)	11	0.030 (0.025-0.034)	12	0.024 (0.021-0.026)	9	0.020 (0.018-0.022)	9	0.022 (0.020-0.024)	9
Chronic respiratory diseases	0.010 (0.009-0.011)	15	0.009 (0.007-0.011)	15	0.009 (0.008-0.011)	15	0.015 (0.012-0.018)	16	0.016 (0.011-0.021)	14	0.016 (0.012-0.019)	15	0.012 (0.009-0.014)	15	0.011 (0.008-0.014)	15	0.011 (0.009-0.014)	15	0.008 (0.007-0.009)	14	0.007 (0.005-0.008)	14	0.008(0.006-0 .008)	14
Diabetes and kidney diseases	0.012 (0.010-0.014)	14	0.012 (0.010-0.014)	13	0.012 (0.011-0.014)	14	0.018 (0.014-0.022)	14	0.016 (0.012-0.020)	15	0.017 (0.013-0.020)	14	0.016 (0.012-0.020)	13	0.016 (0.012-0.021)	13	0.016 (0.013-0.020)	13	0.011 (0.010-0.012)	12	0.010 (0.009-0.011)	11	0.011 (0.010-0.011)	12
Digestive diseases	0.026 (0.020-0.032)	11	0.023 (0.018-0.029)	12	0.025 (0.020-0.030)	11	0.047 (0.032-0.063)	11	0.030 (0.019-0.041)	11	0.039 (0.026-0.051)	11	0.037 (0.026-0.048)	10	0.035 (0.025-0.045)	10	0.036 (0.027-0.046)	10	0.017 (0.015-0.018)	10	0.014 (0.013-0.015)	10	0.015 (0.014-0.017)	10
Enteric infections	0.180 (0.122-0.258)	2	0.199 (0.124-0.292)	1	0.189 (0.131-0.261)	1	0.271 (0.167-0.469)	3	0.268 (0.133-0.449)	2	0.270 (0.171-0.393)	3	0.348 (0.229-0.502)	1	0.396 (0.248-0.580)	1	0.371 (0.253-0.513)	1	0.025 (0.016-0.034)	8	0.020 (0.014-0.031)	8	0.023 (0.016-0.030)	8
HIV/AIDS and sexually transmitted infections	0.003 (0.002-0.004)	16	0.003 (0.002-0.004)	16	0.003 (0.002-0.004)	16	0.022 (0.016-0.030)	13	0.022 (0.016-0.030)	13	0.022 (0.016-0.030)	13	0.001 (0.001-0.002)	17	0.001 (0.001-0.002)	18	0.001 (0.001-0.002)	18	0.001 (0.001-0.002)	16	0.001 (0.001-0.001)	17	0.001 (0.001-0.001)	17
Maternal and neonatal disorders	-	21	0.002 (0.002-0.003)	17	0.001 (0.001-0.001)	18	-	21	0.007 (0.005-0.010)	17	0.004 (0.003-0.005)	17	-	21	0.003 (0.002-0.004)	16	0.001 (0.001-0.002)	17	-	21	0.001 (0.001-0.001)	18	0.000 (0.000-0.001)	19
Mental disorders	0.000 (0.000-0.000)	19	0.000 (0.000-0.000)	20	0.000 (0.000-0.000)	20	0.000 (0.000-0.000)	19	0.000 (0.000-0.000)	20	0.000 (0.000-0.000)	20	0.000 (0.000-0.000)	19	0.000 (0.000-0.000)	20	0.000 (0.000-0.000)	20	0.000 (0.000-0.000)	19	0.000 (0.000-0.000)	20	0.000 (0.000-0.000)	20
Musculoskeletal disorders	0.001 (0.001-0.001)	18	0.001 (0.001-0.002)	19	0.001 (0.001-0.001)	19	0.001 (0.001-0.001)	17	0.001 (0-0.001)	18	0.001 (0.001-0.001)	18	0.000 (0.000-0.001)	18	0.001 (0.000-0.001)	19	0.001 (0.000-0.001)	19	0.001 (0.001-0.001)	17	0.002 (0.001-0.003)	16	0.002 (0.001-0.002)	16
Neglected tropical diseases and malaria	0.122 (0.081-0.182)	4	0.103 (0.066-0.163)	5	0.113 (0.075-0.172)	5	0.370 (0.251-0.513)	2	0.212 (0.132-0.316)	3	0.292 (0.194-0.409)	2	0.208 (0.128-0.332)	4	0.193 (0.121-0.318)	4	0.200 (0.126-0.320)	3	0.01 (0.006-0.024)	13	0.008 (0.005-0.015)	13	0.009 (0.006-0.020)	13
Neoplasms	0.067 (0.053-0.080)	7	0.053 (0.046-0.061)	7	0.060 (0.052-0.069)	7	0.053 (0.036-0.075)	10	0.039 (0.027-0.053)	10	0.046 (0.033-0.062)	10	0.059 (0.042-0.080)	7	0.049 (0.038-0.060)	9	0.054 (0.042-0.068)	8	0.085 (0.070-0.096)	3	0.067 (0.060-0.073)	3	0.076 (0.067-0.083)	3
Neurological disorders	0.013 (0.010-0.015)	13	0.012 (0.008-0.014)	14	0.013 (0.010-0.014)	13	0.016 (0.010-0.021)	15	0.011 (0.007-0.016)	16	0.014 (0.009-0.017)	16	0.015 (0.010-0.018)	14	0.015 (0.010-0.021)	14	0.015 (0.011-0.019)	14	0.013 (0.011-0.014)	11	0.009 (0.008-0.011)	12	0.011 (0.010-0.012)	11
Nutritional deficiencies	0.032 (0.020-0.042)	10	0.040 (0.022-0.066)	9	0.036 (0.024-0.053)	10	0.078 (0.060-0.100)	8	0.073 (0.051-0.104)	8	0.076 (0.059-0.101)	8	0.055 (0.030-0.075)	8	0.074 (0.036-0.130)	6	0.065 (0.039-0.099)	7	0.006 (0.006-0.007)	15	0.006 (0.005-0.007)	15	0.006 (0.006-0.007)	15
Other infectious diseases	0.160 (0.112-0.239)	3	0.175 (0.119-0.256)	2	0.167 (0.116-0.248)	3	0.422 (0.260-0.688)	1	0.415 (0.247-0.657)	1	0.418 (0.258-0.671)	1	0.247 (0.172-0.364)	2	0.284 (0.196-0.413)	2	0.265 (0.185-0.385)	2	0.053 (0.040-0.074)	5	0.050 (0.036-0.072)	5	0.051 (0.039-0.073)	5
Other non-communicable diseases	0.051 (0.039-0.063)	8	0.046 (0.036-0.059)	8	0.049 (0.039-0.059)	8	0.075 (0.045-0.120)	9	0.055 (0.031-0.087)	9	0.065 (0.043-0.097)	9	0.052 (0.036-0.072)	9	0.053 (0.037-0.073)	8	0.053 (0.037-0.070)	9	0.051 (0.043-0.059)	6	0.044 (0.038-0.051)	6	0.048 (0.042-0.054)	6
Respiratory infections and tuberculosis	0.106 (0.090-0.121)	6	0.123 (0.101-0.145)	4	0.114 (0.097-0.132)	4	0.229 (0.177-0.277)	4	0.196 (0.147-0.247)	4	0.213 (0.169-0.259)	4	0.147 (0.119-0.173)	5	0.199 (0.159-0.243)	3	0.172 (0.143-0.205)	5	0.065 (0.053-0.070)	4	0.056 (0.047-0.062)	4	0.061 (0.052-0.065)	4
Self-harm and interpersonal violence	0.042 (0.038-0.045)	9	0.036 (0.033-0.039)	10	0.039 (0.036-0.042)	9	0.152 (0.143-0.164)	6	0.137 (0.129-0.147)	5	0.145 (0.136-0.155)	5	0.030 (0.025-0.035)	11	0.031 (0.026-0.036)	12	0.031 (0.027-0.035)	11	0.040 (0.034-0.043)	7	0.028 (0.026-0.031)	7	0.034 (0.031-0.037)	7
Skin and subcutaneous diseases	0.001 (0.000-0.001)	17	0.001 (0.001-0.002)	18	0.001 (0.001-0.001)	17	0.000 (0.000-0.000)	18	0.000 (0.000-0.000)	19	0.000 (0.000-0.000)	19	0.002 (0.001-0.002)	16	0.002 (0.001-0.003)	17	0.002 (0.001-0.003)	16	0.001 (0.000-0.001)	18	0.001 (0.000-0.001)	19	0.001 (0.000-0.001)	18
Substance use disorders	0.000	20	0.000	21	0.000	21	0.000	20	0.000	21	0.000	21	0.000	20	0.000	21	0.000	21	0.000	20	0.000	21	0.000	21

	(0.000-0.000)		(0.000-0.000)		(0.000-0.000)		(0.000-0.000)		(0.000-0.000)		(0.000-0.000)		(0.000-0.000)		(0.000-0.00)		(0.000-0.000)		(0.000-0.000)		(0.000-0.000)		(0.000-0.000)	
Transport injuries	0.120 (0.106-0.134)	5	0.068 (0.061-0.076)	6	0.095 (0.085-0.105)	6	0.135 (0.099-0.168)	7	0.087 (0.066-0.109)	7	0.111 (0.086-0.137)	7	0.106 (0.085-0.124)	6	0.060 (0.050-0.072)	7	0.084 (0.071-0.098)	6	0.148 (0.136-0.174)	2	0.080 (0.074-0.087)	2	0.115 (0.107-0.128)	2
Unintentional injuries	0.236 (0.204-0.260)	1	0.127 (0.109-0.145)	3	0.183 (0.162-0.201)	2	0.176 (0.140-0.211)	5	0.095 (0.072-0.117)	6	0.136 (0.109-0.162)	6	0.233 (0.181-0.277)	3	0.152 (0.120-0.185)	5	0.194 (0.157-0.227)	4	0.309 (0.285-0.331)	1	0.138 (0.128-0.151)	1	0.226 (0.212-0.240)	1
**2005 year**																								
Cardiovascular diseases	0.019 (0.017-0.021)	12	0.019 (0.017-0.021)	11	0.019 (0.017-0.021)	12	0.027 (0.022-0.031)	13	0.022 (0.018-0.026)	13	0.025 (0.021-0.029)	13	0.021 (0.018-0.024)	12	0.025 (0.021-0.028)	11	0.023 (0.020-0.026)	12	0.017 (0.016-0.018)	8	0.014 (0.013-0.015)	8	0.015 (0.015-0.017)	8
Chronic respiratory diseases	0.007 (0.006-0.008)	16	0.007 (0.005-0.008)	16	0.007 (0.006-0.008)	16	0.012 (0.009-0.014)	16	0.015 (0.011-0.018)	14	0.013 (0.010-0.016)	15	0.008 (0.007-0.009)	16	0.007 (0.006-0.009)	16	0.008 (0.007-0.009)	16	0.005 (0.004-0.005)	15	0.004 (0.004-0.005)	15	0.004 (0.004-0.005)	15
Diabetes and kidney diseases	0.011 (0.001-0.012)	15	0.011 (0.009-0.012)	14	0.011 (0.010-0.012)	14	0.016 (0.013-0.019)	14	0.014 (0.011-0.017)	15	0.015 (0.013-0.018)	14	0.015 (0.013-0.017)	13	0.014 (0.012-0.016)	14	0.014 (0.012-0.017)	14	0.007 (0.007-0.008)	13	0.007 (0.007-0.008)	13	0.007 (0.007-0.008)	13
Digestive diseases	0.021 (0.019-0.024)	11	0.019 (0.016-0.021)	12	0.020 (0.018-0.023)	11	0.040 (0.032-0.048)	12	0.027 (0.021-0.033)	12	0.033 (0.027-0.040)	12	0.029 (0.025-0.034)	9	0.028 (0.023-0.032)	9	0.028 (0.024-0.033)	9	0.010 (0.009-0.011)	11	0.008 (0.008-0.009)	11	0.009 (0.009-0.010)	11
Enteric infections	0.142 (0.099-0.200)	2	0.140 (0.094-0.203)	1	0.141 (0.110-0.194)	1	0.217 (0.157-0.317)	3	0.188 (0.117-0.209)	2	0.202 (0.145-0.278)	2	0.243 (0.166-0.349)	1	0.245 (0.163-0.353)	1	0.244 (0.169-0.339)	1	0.012 (0.008-0.017)	10	0.011 (0.007-0.017)	10	0.012 (0.008-0.016)	10
HIV/AIDS and sexually transmitted infections	0.036 (0.031-0.042)	9	0.037 (0.032-0.043)	9	0.037 (0.031-0.042)	9	0.182 (0.149-0.217)	4	0.183 (0.152-0.216)	3	0.183 (0.151-0.216)	4	0.026 (0.022-0.003)	10	0.027 (0.023-0.031)	10	0.026 (0.023-0.030)	10	0.013 (0.012-0.015)	9	0.013 (0.012-0.014)	9	0.013 (0.012-0.015)	9
Maternal and neonatal disorders	-	21	0.002 (0.002-0.002)	17	0.001 (0.001-0.001)	17	-	21	0.007 (0.005-0.009)	17	0.003 (0.002-0.004)	17	-	21	0.002 (0.002-0.003)	17	0.001 (0.001-0.001)	18	-	21	0.001 (0.001-0.001)	18	0.000 (0.000-0.000)	19
Mental disorders	0.000 (0.000-0.000)	20	0.000 (0.000-0.000)	20	0.000 (0.000-0.000)	21	0.000 (0.000-0.000)	19	0.000 (0.000-0.000)	20	0.000 (0.000-0.000)	20	0.000 (0.000-0.000)	19	0.000 (0.000-0.000)	20	0.000 (0.000-0.000)	20	0.000 (0.000-0.000)	20	0.000 (0.000-0.000)	20	0.000 (0.000-0.000)	21
Musculoskeletal disorders	0.001 (0.001-0.001)	18	0.001 (0.001-0.001)	18	0.001 (0.001-0.001)	19	0.001 (0.001-0.001)	17	0.001 (0.000-0.001)	18	0.001 (0.001-0.001)	18	0.000 (0.000-0.001)	18	0.001 (0.000-0.001)	19	0.001 (0.000-0.001)	19	0.001 (0.001-0.001)	17	0.002 (0.002-0.003)	17	0.002 (0.001-0.002)	17
Neglected tropical diseases and malaria	0.077 (0.051-0.114)	5	0.060 (0.039-0.09)	5	0.069 (0.045-0.101)	6	0.231 (0.147-0.347)	1	0.148 (0.089-0.226)	5	0.190 (0.120-0.287)	3	0.107 (0.069-0.163)	4	0.090 (0.058-0.133)	5	0.099 (0.063-0.147)	5	0.006 (0.003-0.015)	14	0.004 (0.002-0.009)	14	0.005 (0.003-0.012)	14
Neoplasms	0.055 (0.047-0.064)	7	0.044 (0.039-0.048)	7	0.049 (0.044-0.055)	7	0.050 (0.039-0.065)	9	0.040 (0.032-0.050)	10	0.045 (0.037-0.056)	9	0.054 (0.043-0.067)	7	0.043 (0.036-0.051)	8	0.049 (0.040-0.058)	7	0.064 (0.058-0.071)	3	0.051 (0.047-0.053)	3	0.058 (0.054-0.061)	3
Neurological disorders	0.011 (0.009-0.013)	14	0.010 (0.008-0.011)	15	0.011 (0.009-0.012)	15	0.014 (0.011-0.017)	15	0.010 (0.007-0.013)	16	0.012 (0.010-0.014)	16	0.013 (0.010-0.016)	15	0.012 (0.010-0.015)	15	0.013 (0.010-0.015)	15	0.010 (0.009-0.010)	12	0.007 (0.007-0.008)	12	0.009 (0.008-0.009)	12
Nutritional deficiencies	0.012 (0.010-0.014)	13	0.015 (0.012-0.018)	13	0.013 (0.011-0.015)	13	0.043 (0.033-0.053)	11	0.042 (0.032-0.054)	9	0.043 (0.034-0.051)	10	0.014 (0.011-0.017)	14	0.019 (0.015-0.023)	13	0.016 (0.013-0.019)	13	0.003 (0.003-0.003)	16	0.003 (0.003-0.003)	16	0.003 (0.003-0.003)	16
Other infectious diseases	0.105 (0.079-0.143)	3	0.110 (0.080-0.152)	2	0.107 (0.08-0.146)	3	0.217 (0.154-0.312)	2	0.212 (0.147-0.305)	1	0.215 (0.150-0.304)	1	0.158 (0.117-0.216)	3	0.170 (0.121-0.235)	2	0.163 (0.121-0.225)	3	0.019 (0.017-0.023)	7	0.018 (0.016-0.021)	7	0.019 (0.017-0.022)	7
Other non-communicable diseases	0.043 (0.037-0.050)	8	0.040 (0.034-0.047)	8	0.041 (0.036-0.048)	8	0.067 (0.045-0.100)	8	0.053 (0.034-0.076)	8	0.060 (0.043-0.085)	8	0.046 (0.038-0.058)	8	0.045 (0.037-0.055)	6	0.046 (0.038-0.055)	8	0.037 (0.035-0.041)	4	0.034 (0.031-0.036)	4	0.036 (0.034-0.038)	4
Respiratory infections and tuberculosis	0.072 (0.063-0.081)	6	0.081 (0.067-0.093)	4	0.076 (0.066-0.086)	4	0.163 (0.132-0.191)	5	0.153 (0.122-0.185)	4	0.158 (0.133-0.184)	5	0.094 (0.079-0.108)	5	0.115 (0.094-0.135)	4	0.104 (0.088-0.120)	4	0.032 (0.030-0.034)	6	0.029 (0.026-0.030)	5	0.030 (0.029-0.032)	5
Self-harm and interpersonal violence	0.029(0.026-0.031)	10	0.021(0.02-0.023)	10	0.025 (0.023-0.027)	10	0.048 (0.043-0.054)	10	0.036 (0.033-0.039)	11	0.042 (0.038-0.046)	11	0.025 (0.02-0.029)	11	0.021 (0.018-0.024)	12	0.023 (0.020-0.026)	11	0.033 (0.031-0.035)	5	0.021 (0.02-0.022)	6	0.027 (0.026-0.029)	6
Skin and subcutaneous diseases	0.001 (0.000-0.001)	17	0.001 (0.000-0.001)	19	0.001 (0.001-0.001)	18	0.000 (0.000-0.000)	18	0.000 (0.000-0.000)	19	0.000 (0.000-0.000)	19	0.001 (0.001-0.002)	17	0.002 (0.001-0.002)	18	0.002 (0.001-0.002)	17	0.000 (0.000-0.001)	18	0.000 (0.000-0.000)	19	0.000 (0.000-0.001)	18
Substance use disorders	0.000 (0.000-0.000	19	0.000 (0.000-0.000)	21	0.000 (0.000-0.000)	20	0.000 (0.000-0.000)	20	0.000 (0.000-0.000)	21	0.000 (0.000-0.000)	21	0.000 (0.000-0.000)	20	0.000 (0.000-0.000)	21	0.000 (0.000-0.000)	21	0.000 (0.000-0.000)	19	0.000 (0.000-0.000)	21	0.000 (0.000-0.000)	20
Transport injuries	0.088 (0.075-0.098)	4	0.05 (0.045-0.055)	6	0.070 (0.062-0.076)	5	0.121 (0.091-0.144)	7	0.076 (0.061-0.094)	7	0.099 (0.080-0.117)	7	0.081 (0.065-0.093)	6	0.045 (0.038-0.051)	7	0.063 (0.054-0.072)	6	0.105 (0.096-0.114)	2	0.059 (0.056-0.062)	2	0.083 (0.078-0.088)	2
Unintentional injuries	0.169 (0.148-0.186)	1	0.096 (0.084-0.106)	3	0.134 (0.119-0.146)	2	0.148 (0.123-0.180)	6	0.082 (0.068-0.096)	6	0.115 (0.098-0.135)	6	0.197 (0.165-0.224)	2	0.132 (0.111-0.151)	3	0.166 (0.142-0.187)	2	0.182 (0.165-0.192)	1	0.074 (0.071-0.078)	1	0.130 (0.121-0.137)	1

**2019 year**																								
Cardiovascular diseases	0.014 (0.012-0.017)	12	0.014 (0.012-0.016)	12	0.014 (0.012-0.016)	12	0.021 (0.017-0.029)	12	0.018 (0.014-0.022)	13	0.02 (0.015-0.025)	13	0.015 (0.013-0.019)	11	0.018 (0.015-0.021)	10	0.017 (0.014-0.020)	11	0.010 (0.009-0.011)	9	0.008 (0.008-0.009)	9	0.009 (0.008-0.010)	9
Chronic respiratory diseases	0.004 (0.004-0.005)	16	0.005 (0.004-0.006)	16	0.005 (0.004-0.005)	16	0.008 (0.006-0.010)	16	0.011 (0.007-0.015)	15	0.009 (0.007-0.012)	16	0.005 (0.004-0.006)	16	0.005 (0.004-0.006)	16	0.005 (0.004-0.006)	16	0.002 (0.002-0.003)	15	0.002 (0.002-0.003)	14	0.002 (0.002-0.003)	15
Diabetes and kidney diseases	0.009 (0.008-0.010)	13	0.008 (0.007-0.010)	13	0.009 (0.007-0.01)	13	0.014 (0.011-0.018)	14	0.012 (0.009-0.014)	14	0.013 (0.010-0.016)	14	0.011 (0.009-0.013)	13	0.011 (0.009-0.013)	13	0.011 (0.009-0.013)	13	0.004 (0.004-0.005)	13	0.005 (0.004-0.005)	13	0.004 (0.004-0.005)	13
Digestive diseases	0.016 (0.013-0.021)	11	0.014 (0.011-0.017)	11	0.015 (0.012-0.019)	11	0.032 (0.024-0.041)	11	0.021 (0.017-0.027)	12	0.027 (0.021-0.034)	11	0.020 (0.016-0.026)	10	0.019 (0.015-0.024)	9	0.020 (0.015-0.025)	10	0.006 (0.005-0.007)	12	0.005 (0.005-0.006)	12	0.006 (0.005-0.006)	12
Enteric infections	0.098 (0.069-0.141)	1	0.090 (0.058-0.133)	1	0.094 (0.067-0.131)	1	0.165 (0.112-0.252)	1	0.117 (0.070-0.198)	1	0.141 (0.098-0.201)	1	0.147 (0.097-0.216)	1	0.143 (0.092-0.205)	1	0.145 (0.101-0.205)	1	0.007 (0.005-0.01)	11	0.006 (0.004-0.009)	10	0.006 (0.004-0.009)	11
HIV/AIDS and sexually transmitted infections	0.022 (0.018-0.026)	10	0.022 (0.018-0.025)	9	0.022 (0.018-0.026)	9	0.075 (0.059-0.093)	7	0.076 (0.060-0.094)	5	0.076 (0.060-0.094)	6	0.014 (0.012-0.017)	12	0.014 (0.011-0.016)	12	0.014 (0.012-0.017)	12	0.015 (0.013-0.017)	7	0.013 (0.012-0.015)	7	0.014 (0.012-0.016)	7
Maternal and neonatal disorders	-	21	0.002 (0.001-0.002)	17	0.001 (0.001-0.001)	17	-	21	0.005 (0.003-0.007)	17	0.002 (0.002-0.003)	17	-	21	0.002 (0.001-0.002)	17	0.001 (0.001-0.001)	18	-	21	0.000 (0.000-0.000)	19	0.000 (0.000-0.000)	19
Mental disorders	0.000 (0.000-0.000)	19	0.000 (0.000-0.000)	20	0.000 (0.000-0.000)	20	0.000 (0.000-0.000)	19	0.000 (0.000-0.000)	20	0.000 (0.000-0.000)	20	0.000 (0.000-0.000)	19	0.000 (0.000-0.000)	20	0.000 (0.000-0.000)	20	0.000 (0.000-0.000)	19	0.000 (0.000-0.000)	20	0.000 (0.000-0.000)	20
Musculoskeletal disorders	0.000 (0.000-0.001)	18	0.001 (0.001-0.001)	18	0.001 (0.001-0.001)	18	0.001 (0.000-0.001)	17	0.001 (0.001-0.001)	18	0.001 (0.001-0.001)	18	0.000 (0.000-0.000)	18	0.001 (0.000-0.001)	19	0.001 (0-0.001)	19	0.001 (0.001-0.001)	17	0.002 (0.001-0.002)	16	0.001 (0.001-0.002)	17
Neglected tropical diseases and malaria	0.046 (0.028-0.071)	6	0.035 (0.021-0.055)	6	0.041 (0.025-0.063)	7	0.125 (0.067-0.212)	2	0.078 (0.039-0.136)	4	0.102 (0.054-0.172)	3	0.055 (0.034-0.083)	5	0.046 (0.028-0.071)	5	0.050 (0.031-0.077)	5	0.003 (0.002-0.008)	14	0.002 (0.001-0.004)	15	0.003 (0.001-0.006)	14
Neoplasms	0.046 (0.040-0.053)	5	0.038 (0.033-0.043)	5	0.042 (0.038-0.047)	6	0.046 (0.036-0.056)	9	0.039 (0.031-0.048)	9	0.042 (0.034-0.051)	9	0.048 (0.040-0.057)	7	0.039 (0.032-0.046)	6	0.044 (0.037-0.051)	6	0.051 (0.044-0.057)	3	0.040 (0.037-0.044)	1	0.046 (0.041-0.050)	2
Neurological disorders	0.008 (0.007-0.010)	14	0.008 (0.006-0.009)	15	0.008 (0.007-0.009)	14	0.011 (0.009-0.015)	15	0.009 (0.007-0.011)	16	0.010 (0.008-0.013)	15	0.009 (0.007-0.012)	14	0.009 (0.007-0.011)	14	0.009 (0.007-0.011)	14	0.007 (0.006-0.008)	10	0.006 (0.005-0.006)	11	0.006 (0.006-0.007)	10
Nutritional deficiencies	0.006 (0.005-0.007)	15	0.008 (0.006-0.010)	14	0.007 (0.005-0.008)	15	0.018 (0.014-0.024)	13	0.023 (0.017-0.031)	10	0.021 (0.016-0.027)	12	0.005 (0.004-0.007)	15	0.008 (0.006-0.010)	15	0.006 (0.005-0.008)	15	0.002 (0.001-0.002)	16	0.002 (0.001-0.002)	17	0.002 (0.001-0.002)	16
Other infectious diseases	0.049 (0.039-0.062)	4	0.049 (0.037-0.063)	3	0.049 (0.039-0.062)	3	0.102 (0.073-0.144)	4	0.104 (0.071-0.149)	2	0.103 (0.073-0.148)	2	0.064 (0.050-0.079)	3	0.062 (0.047-0.078)	3	0.063 (0.050-0.078)	3	0.010 (0.009-0.012)	8	0.009 (0.008-0.011)	8	0.010 (0.009-0.011)	8
Other non-communicable diseases	0.035 (0.029-0.042)	8	0.033 (0.028-0.039)	8	0.034 (0.029-0.040)	8	0.057 (0.039-0.083)	8	0.046 (0.031-0.066)	8	0.051 (0.038-0.070)	8	0.037 (0.030-0.046)	8	0.038 (0.030-0.045)	7	0.038 (0.031-0.045)	8	0.026 (0.023-0.029)	4	0.024 (0.021-0.026)	4	0.025 (0.023-0.027)	4
Respiratory infections and tuberculosis	0.044 (0.037-0.051)	7	0.046 (0.038-0.055)	4	0.045 (0.038-0.052)	5	0.096 (0.076-0.120)	5	0.095 (0.075-0.117)	3	0.095 (0.078-0.116)	4	0.052 (0.043-0.061)	6	0.057 (0.046-0.069)	4	0.054 (0.046-0.065)	4	0.015 (0.014-0.017)	6	0.014 (0.013-0.015)	5	0.015 (0.013-0.016)	6
Self-harm and interpersonal violence	0.023 (0.020-0.026)	9	0.016 (0.014-0.018)	10	0.020 (0.017-0.022)	10	0.035 (0.028-0.042)	10	0.022 (0.019-0.026)	11	0.029 (0.024-0.034)	10	0.023 (0.019-0.027)	9	0.017 (0.015-0.020)	11	0.020 (0.017-0.023)	9	0.021 (0.02-0.024)	5	0.014 (0.013-0.015)	6	0.018 (0.016-0.019)	5
Skin and subcutaneous diseases	0.001 (0.000-0.001)	17	0.001 (0.000-0.001)	19	0.001 (0.000-0.001)	19	0.000 (0.000-0.000)	18	0.000 (0.000-0.000)	19	0.000 (0.000-0.000)	19	0.001 (0.000-0.001)	17	0.001 (0.001-0.002)	18	0.001 (0.001-0.001)	17	0.000 (0.000-0.001)	18	0.000 (0.000-0.000)	18	0.000 (0.000-0.000)	18
Substance use disorders	0.000 (0.000-0.000)	20	0.000 (0.000-0.000)	21	0.000 (0.000-0.000)	21	0.000 (0.000-0.000)	20	0.000 (0.000-0.000)	21	0.000 (0.000-0.000)	21	0.000 (0.000-0.000)	20	0.000 (0.000-0.000)	21	0.000 (0.000-0.000)	21	0.000 (0.000-0.000)	20	0.000 (0.000-0.000)	21	0.000 (0.000-0.000)	21
Transport injuries	0.057 (0.047-0.067)	3	0.033 (0.029-0.039)	7	0.045 (0.039-0.053)	4	0.087 (0.065-0.113)	6	0.056 (0.043-0.077)	7	0.071 (0.056-0.093)	7	0.055 (0.044-0.067)	4	0.031 (0.025-0.037)	8	0.043 (0.037-0.052)	7	0.056 (0.050-0.062)	2	0.033 (0.030-0.036)	3	0.045 (0.041-0.049)	3
Unintentional injuries	0.089 (0.075-0.104)	2	0.052 (0.042-0.061)	2	0.071 (0.060-0.082)	2	0.107 (0.084-0.141)	3	0.061 (0.048-0.075)	6	0.084 (0.068-0.105)	5	0.097 (0.078-0.118)	2	0.063 (0.049-0.079)	2	0.081 (0.065-0.097)	2	0.091 (0.082-0.099)	1	0.039 (0.037-0.042)	2	0.066 (0.061-0.072)	1

Schedule3 Cause-deleted life expectancy (LE) and life expectancy loss at aged 5-14 years in region-specific from 1990 to 2019

	1990 year								2005 year								2019 year							
	Global		LICs		Lower MICs		Upper MICs		Global		LICs		Lower MICs		Upper MICs		Global		LICs		Lower MICs		Upper MICs	
**Cause of death**	cause-eliminated LE	LE Loss (year)	cause-eliminated LE	LE Loss (year)	cause-eliminated LE	LE Loss (year)	cause-eliminated LE	LE Loss (year)	cause-eliminated LE	LE Loss (year)	cause-eliminated LE	LE Loss (year)	cause-eliminated LE	LE Loss (year)	cause-eliminated LE	LE Loss (year)	cause-eliminated LE	LE Loss (year)	cause-eliminated LE	LE Loss (year)	cause-eliminated LE	LE Loss (year)	cause-eliminated LE	LE Loss (year)
Enteric infections	66.658	0.117	58.814	0.145	63.403	0.217	67.179	0.014	68.335	0.089	59.496	0.110	65.174	0.147	68.762	0.007	71.440	0.063	64.893	0.085	68.212	0.092	72.445	0.004
Unintentional injuries	66.654	0.113	58.742	0.073	63.300	0.113	67.306	0.140	68.330	0.085	59.449	0.063	65.127	0.099	68.838	0.083	71.424	0.047	64.859	0.050	68.171	0.051	72.485	0.045
Other infectious diseases	66.645	0.103	58.894	0.225	63.341	0.154	67.197	0.032	68.313	0.068	59.503	0.117	65.126	0.098	68.767	0.012	71.410	0.033	64.870	0.062	68.160	0.040	72.447	0.007
Respiratory infections and tuberculosis	66.612	0.070	58.783	0.114	63.287	0.100	67.203	0.038	68.293	0.048	59.472	0.086	65.090	0.063	68.774	0.019	71.407	0.030	64.866	0.057	68.154	0.034	72.450	0.010
Neglected tropical diseases and malaria	66.611	0.070	58.826	0.157	63.304	0.117	67.171	0.006	68.289	0.044	59.490	0.104	65.087	0.059	68.758	0.003	71.404	0.027	64.869	0.061	68.152	0.032	72.442	0.002
Transport injuries	66.600	0.058	58.728	0.060	63.236	0.049	67.236	0.071	68.289	0.044	59.440	0.054	65.065	0.038	68.808	0.053	71.407	0.030	64.851	0.043	68.148	0.027	72.471	0.030
Neoplasms	66.579	0.037	58.694	0.025	63.218	0.031	67.212	0.047	68.276	0.031	59.411	0.025	65.057	0.029	68.792	0.037	71.405	0.028	64.834	0.025	68.148	0.028	72.471	0.031
Other non-communicable diseases	66.572	0.030	58.704	0.035	63.217	0.031	67.195	0.030	68.271	0.026	59.419	0.033	65.055	0.027	68.778	0.023	71.400	0.023	64.839	0.031	68.144	0.024	72.457	0.017
Self-harm and interpersonal violence	66.566	0.024	58.747	0.078	63.205	0.018	67.186	0.021	68.261	0.016	59.409	0.023	65.041	0.014	68.772	0.017	71.390	0.013	64.826	0.017	68.133	0.013	72.452	0.012
Nutritional deficiencies	66.564	0.022	58.709	0.041	63.224	0.038	67.169	0.004	68.254	0.008	59.409	0.023	65.037	0.010	68.757	0.002	71.382	0.004	64.821	0.012	68.124	0.004	72.441	0.001
Digestive diseases	66.557	0.015	58.689	0.021	63.208	0.021	67.175	0.010	68.258	0.013	59.404	0.018	65.044	0.017	68.761	0.006	71.387	0.010	64.825	0.016	68.133	0.012	72.444	0.004
Cardiovascular diseases	66.557	0.015	58.686	0.017	63.204	0.017	67.179	0.014	68.257	0.012	59.400	0.013	65.041	0.014	68.765	0.010	71.386	0.009	64.820	0.012	68.131	0.010	72.447	0.006
Neurological disorders	66.549	0.008	58.676	0.007	63.196	0.009	67.172	0.007	68.252	0.007	59.393	0.007	65.035	0.008	68.760	0.005	71.383	0.005	64.815	0.006	68.126	0.006	72.445	0.004
Diabetes and kidney diseases	66.549	0.008	58.678	0.009	63.196	0.009	67.172	0.007	68.252	0.007	59.394	0.008	65.036	0.009	68.760	0.005	71.383	0.006	64.816	0.008	68.127	0.007	72.443	0.003
Chronic respiratory diseases	66.547	0.006	58.677	0.008	63.194	0.007	67.170	0.005	68.249	0.004	59.393	0.007	65.032	0.005	68.758	0.003	71.380	0.003	64.814	0.006	68.123	0.003	72.442	0.002
HIV/AIDS and sexually transmitted infections	66.543	0.002	58.681	0.012	63.188	0.001	67.166	0.001	68.268	0.023	59.486	0.099	65.043	0.016	68.763	0.008	71.392	0.014	64.854	0.045	68.129	0.009	72.450	0.009
Skin and subcutaneous diseases	66.542	0.001	58.669	0.000	63.188	0.001	67.166	0.000	68.246	0.001	59.386	0.000	65.028	0.001	68.755	0.000	71.378	0.000	64.809	0.000	68.121	0.001	72.441	0.000
Maternal and neonatal disorders	66.542	0.001	58.671	0.002	63.188	0.001	67.165	0.000	68.246	0.001	59.388	0.002	65.028	0.001	68.755	0.000	71.378	0.001	64.810	0.001	68.121	0.000	72.441	0.000
Musculoskeletal disorders	66.542	0.001	58.669	0.000	63.187	0.000	67.166	0.001	68.246	0.001	59.387	0.000	65.028	0.000	68.756	0.001	71.378	0.000	64.809	0.000	68.120	0.000	72.441	0.001
Mental disorders	66.542	0.000	58.669	0.000	63.187	0.000	67.165	0.000	68.245	0.000	59.386	0.000	65.027	0.000	68.755	0.000	71.377	0.000	64.808	0.000	68.120	0.000	72.440	0.000
Substance use disorders	66.542	0.000	58.669	0.000	63.187	0.000	67.165	0.000	68.245	0.000	59.386	0.000	65.027	0.000	68.755	0.000	71.377	0.000	64.808	0.000	68.120	0.000	72.440	0.000
